# Autophagy is induced by swine acute diarrhea syndrome coronavirus through the cellular IRE1-JNK-Beclin 1 signaling pathway after an interaction of viral membrane-associated papain-like protease and GRP78

**DOI:** 10.1371/journal.ppat.1011201

**Published:** 2023-03-08

**Authors:** Da Shi, Ling Zhou, Hongyan Shi, Jiyu Zhang, Jialin Zhang, Liaoyuan Zhang, Dakai Liu, Tingshuai Feng, Miaomiao Zeng, Jianfei Chen, Xin Zhang, Mei Xue, Zhaoyang Jing, Jianbo Liu, Zhaoyang Ji, Haojie He, Longjun Guo, Yang Wu, Jingyun Ma, Li Feng

**Affiliations:** 1 State Key Laboratory of Veterinary Biotechnology, Harbin Veterinary Research Institute, Chinese Academy of Agricultural Sciences, Xiangfang District, China; 2 College of Animal Science, South China Agricultural University, Tianhe District, China; Oklahoma State University College of Veterinary Medicine, UNITED STATES

## Abstract

Autophagy plays an important role in the infectious processes of diverse pathogens. For instance, cellular autophagy could be harnessed by viruses to facilitate replication. However, it is still uncertain about the interplay of autophagy and swine acute diarrhea syndrome coronavirus (SADS-CoV) in cells. In this study, we reported that SADS-CoV infection could induce a complete autophagy process both *in vitro* and *in vivo*, and an inhibition of autophagy significantly decreased SADS-CoV production, thus suggesting that autophagy facilitated the replication of SADS-CoV. We found that ER stress and its downstream IRE1 pathway were indispensable in the processes of SADS-CoV-induced autophagy. We also demonstrated that IRE1-JNK-Beclin 1 signaling pathway, neither PERK-EIF2S1 nor ATF6 pathways, was essential during SADS-CoV-induced autophagy. Importantly, our work provided the first evidence that expression of SADS-CoV PLP2-TM protein induced autophagy through the IRE1-JNK-Beclin 1 signaling pathway. Furthermore, the interaction of viral PLP2-TM^F451-L490^ domain and substrate-binding domain of GRP78 was identified to activate the IRE1-JNK-Beclin 1 signaling pathway, and thus resulting in autophagy, and in turn, enhancing SADS-CoV replication. Collectively, these results not only showed that autophagy promoted SADS-CoV replication in cultured cells, but also revealed that the molecular mechanism underlying SADS-CoV-induced autophagy in cells.

## Author summary

SADS-CoV, a novel bat-origin pathogen belonging to the family Alphacoronavirus, was confirmed to be the aetiological agent that caused mass death of piglets in southern China in 2017. Strikingly, since the outbreak of SADS-CoV, multiple studies have highlighted the risk of cross-species transmission due to its broad cell tropism. A better understanding of the pathogenic mechanisms is expected to result in a more specific SADS-CoV prevention and control measures. As yet, the pathogenic mechanisms of SADS-CoV have so far not been characterized well. Here, we found that SADS-CoV infection induced a complete autophagy process *in vitro* and *in vivo* to facilitate virus propagation. Furthermore, it’s indicated that ER stress and IRE1 pathway played indispensable functions in SADS-CoV-induced autophagy. Notably, we have provided first evidence that SADS-CoV PLP2-TM^F451-L490^ domain induced autophagy by interacting with substrate-binding domain of GRP78 to activate the IRE1-JNK-Beclin 1 signaling pathway. Overall, our study presented detailed mechanism of SADS-CoV-induced autophagy as well as a contribution for SADS-CoV pathogenesis.

## Introduction

The outbreaks of severe diarrhea occurred in suckling piglets of various swine herds in February 2017 throughout Guangdong Province, China [1]. However, it was difficult to elucidate the precise pathogenesis, owing to the clinical signs and pathological lesions of swine acute diarrhea syndrome coronavirus (SADS-CoV) infection in pigs were similar to those caused by porcine epidemic diarrhea virus (PEDV), transmissible gastroenteritis virus (TGEV), and porcine deltacoronavirus (PDCoV), making the diagnosis indistinguishable [1]. The causative enteric pathogen was identified as a novel porcine CoV, which belongs to the species *Rhinolophus bat coronavirus HKU2* [1], and later on was named SADS-CoV. In addition, the SADS-CoV was also named as swine enteric alphacoronavirus (SeACoV) [1] or porcine enteric alphacoronavirus (PEAV) [2]. Despite the fact that there hasn’t been a SADS-CoV epidemic in China since 2019, the evidence revealed that SADS-CoV has not vanished [3]. SADS-CoV showed a wide range of cell tropism and can proliferate in multiple vertebrate cells, including human, mouse, avian, bat, mink, pig, canine, monkey, and others [4]. Strinkingly, SADS-CoV can infect the hamster kidney cell line BHK-21, which is not susceptible to other known swine enteric CoVs, including TGEV, PEDV and PDCoV [5] as well as two human CoVs, severe acute respiratory syndrome coronavirus-1 (SARS-CoV-1) and middle east respiratory syndrome coronavirus (MERS-CoV) [6,7].

Coronaviruses, a member of the order Nidovirales, are enveloped, positive-sense, and single-stranded RNA viruses [8]. Their genomes are the largest among all known RNA viruses, ranging from approximately 26 to 32 kb. Coronaviruses can induce the formation of double membrane vesicles (DMVs), which are associated with viral replication/transcription complexes (RTCs), after invading the cells [9,10]. The morphological similarity of autophagosomes and coronavirus-induced DMVs has led to some early speculations that cellular autophagy machineries may be utilized by coronaviruses to promote viral replication.

Autophagy is a dynamic and conserved eukaryotic process that functions to clean out damaged protein aggregates and obsolete organelles. This process targets cellular debris to lysosomes for degradation through autophagosomes with single- or double-membrane vesicle structures [11,12]. In yeast, more than thirty specific genes that participate in autophagy pathway have been identified [13]. During autophagy, MAP1LC3/LC3 (microtubule-associated protein 1 light chain 3) is conjugated to phosphatidylethanolamine to form lipidated LC3-II, which is used as an autophagosomal marker in host cells. The multifunctional polyubiquitin binding protein p62/SQSTM1 serves as a substrate for autophagic degradation and can be used to assess autophagic degradation and autophagic flux [14]. Previous studies showed that autophagy not only served a protective function in cell survival under stress, but also played a role in pathogen infection [15]. The pathogens herpes simplex virus type 1 and human immunodeficiency virus type 1 can inhibit autophagy to facilitate viral replication [16,17]. Furthermore, autophagy can restrict the replication of intracellular pathogens, such as tobacco mosaic virus and Sindbis virus, during infection processes [18,19]. An understanding of the details between autophagy and viral infection is critical for controlling disease transmission.

Endoplasmic reticulum (ER) is the major cellular organelle for protein synthesis, folding, modification, and trafficking [20]. However, several endogenous imbalances in cells can contribute to an ER malfunction, which is known as ER stress [21]. The cells have evolved an adaptive strategy by utilizing the unfolded protein response (UPR) pathway, through which unfolded/misfolded proteins could be refolded or degraded to maintain ER homeostasis. In mammalian cells, the UPR is composed of three pathways that are initiated by distinct ER sensors, including inositol-requiring enzyme 1 (IRE1), protein kinase RNA (PKR)-like ER kinase (PERK), and activating transcription factor-6 (ATF6) [22]. These sensors are usually in an inactive state induced by immunoglobulin binding protein (Bip)/glucose-regulated protein of 78 kDa (GRP78). Under conditions of ER stress, GRP78 is released from the ER sensors, leading to an activation of the UPR. IRE1 and PERK homodimerize upon release of GRP78 and undergo autophosphorylation. ATF6 is transported to the Golgi, where it is proteolytically cleaved. An activation of each senor produces an active transcription factor, which in turn activates downstream target genes to restore ER homeostasis. The UPR is also involved in the pathogenesis of several bacteria and viruses, such as group A *Streptococcus* [23], Mycobacterium tuberculosis [24], Japanese encephalitis virus [25], hepatitis C virus [26], and influenza A virus [27]. These pathogens modulate individual pathway of the UPR in distinct ways to enable their replication in host cells. Recent data suggest that the disturbance of UPR signal branches may play a role in developing and progressing various human diseases. The ER stress inducer thapsigargin efficiently inhibits coronavirus (SARS-CoV-2, HcoV-229E, TGEV, MERS-CoV) replication in different cell types [28–30] Additionally, ER stress can trigger autophagy through the activation of UPR components [31,32], and several viruses of the coronavirus family have been reported to activate autophagy that involved in viral replication [33]. These findings promote us to investigate the interplay and molecular mechanisms between SADS-CoV infection and the activation of autophagy.

In this study, the molecular mechanism of SADS-CoV-induced autophagy was investigated. The results provided the first evidence that SADS-CoV activated the IRE1-JNK-Beclin 1 pathway in infected cells. The membrane-associated papain-like protease PLP2 (PLP2-TM) in the nonstructural protein (nsp) 3 of SADS-CoV was identified to be one mechanism of the induction of autophagy. Expression of PLP2-TM alone was sufficient to induce autophagy in a variety of cells. We also found that the interaction of PLP2-TM and GRP78 activated the IRE1-JNK-Beclin 1 pathway, and then induced autophagy. Taken together, this study provided novel insights into the coronavirus-induced autophagy that was modulated by cellular factors. Moreover, the current data suggested that intricate cross-talk between multiple cellular signaling pathways contributed to the host responses during coronaviral infection.

## Results

### SADS-CoV infection activates autophagy in the cultured cells

**Fig 1:**
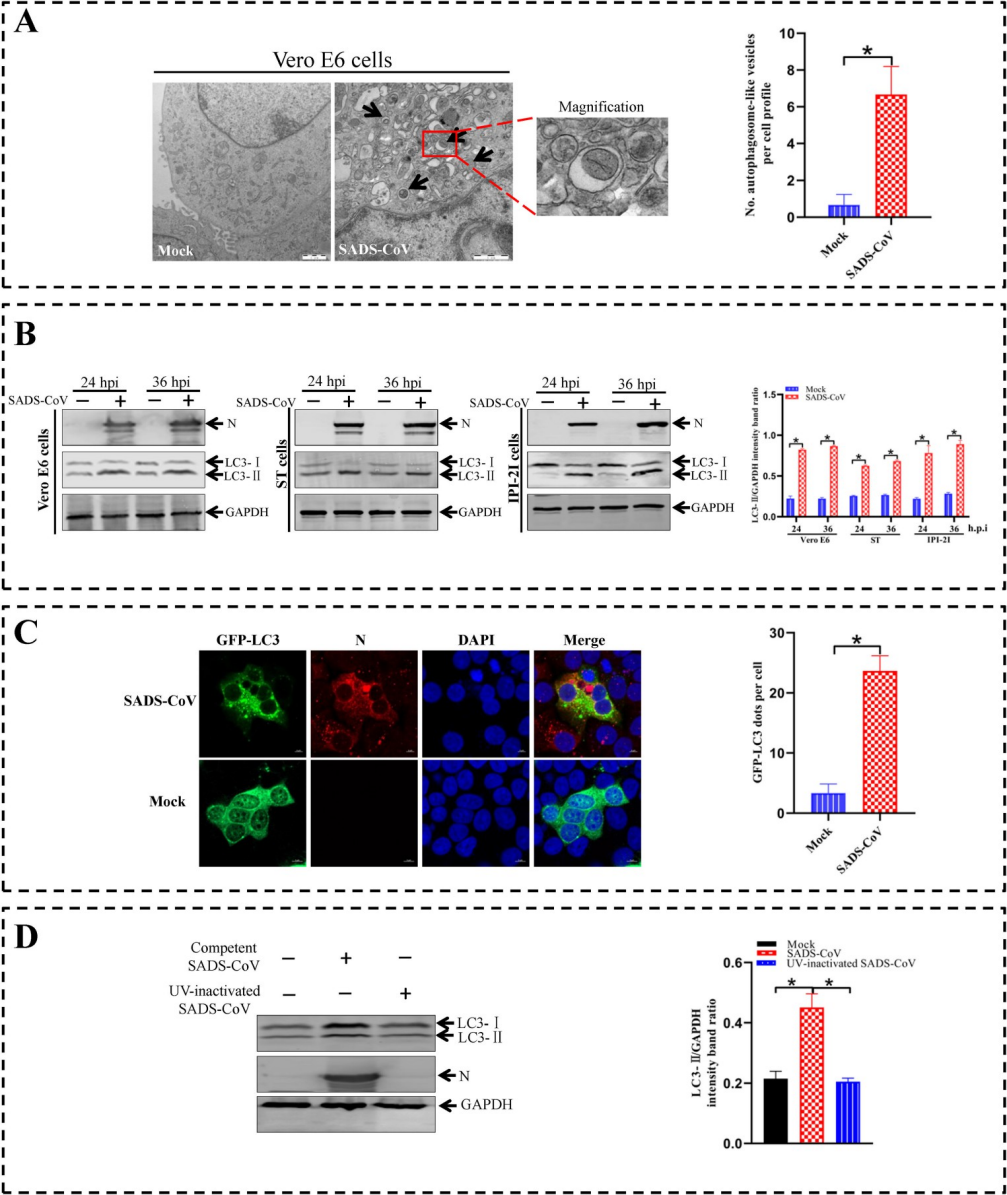
SADS-CoV infection induces autophagy in cultured cells. (**A**) Vero E6 cells are mock-infected or infected with SADS-CoV for 36 h, and then processed for an observation with transmission electron microscopy (TEM). Black arrows indicate characteristics of single- or double-membrane vesicles. Scale bars indicate 1 μm. (**B**) Vero E6, ST and IPI-2I cells are mock-infected or infected with SADS-CoV at the indicated time points (24 and 36 hpi). Cellular lysates are analyzed by western blotting with anti-LC3 and anti-viral N antibodies. GAPDH is used as a protein loading control. (**C**) Vero E6 cells transfected with pGFP-LC3 plasmids for 24 h are infected with SADS-CoV, and then subjected to a confocal immunofluorescence analysis at 36 hpi. The puncta of GFP-LC3 (green) are counted in viral N protein (red) positive cells. (**D**) Vero E6 cells are inoculated with competent SADS-CoV or UV-inactivated SADS-CoV for 36 h. Subsequently, cellular lysates are analyzed as described in B. Means and SD (error bars) of three independent experiments are indicated (*P < 0.05).

Transmission electron microscopy (TEM) is an accepted standard method for observing the formation of single- or double membrane autophagic compartments in the perinuclear region, and evaluating the morphology of autophagic compartments [34]. Thus, to determine whether autophagy was triggered upon SADS-CoV infection, TEM was employed to perform an ultrastructural analysis of SADS-CoV-infected Vero E6 cells. A significant increase of the number of single- or double-membrane vesicles in the perinuclear region was observed in the SADS-CoV-infected cells. In addition, recognizable cytoplasmic contents or degraded organelles were sequestered in the homogeneously sized vesicles with a typical morphology of autophagic vacuoles. In contrast, similar vesicles were rarely observed in the uninfected (mock-infected) cells. These uninfected cells exhibited an extremely dense cytoplasm and contained many morphologically normal organelles (Fig 1A). Taken together, these data demonstrated that SADS-CoV infection induced autophagosome-like vesicles in the Vero E6 cells.

To further verify if the observed vesicles were related to autophagy in the SADS-CoV-infected cells, an immunoblotting analysis was performed to examine the conversion of endogenous LC3 protein, which is an important diagnostic marker of autophagy [14]. Upon autophagy induction, a series of conjugation reactions led to the conversion of cytosolic microtubule-associated LC3 (LC3-I) to the lipidated form LC3 (LC3-II). The amount of LC3-II was correlated with the number of autophagosomes [35]. Therefore, LC3 conversion was examined by western blotting using an anti-LC3 antibody that recognized both forms of LC3 during SADS-CoV infection. The monoclonal antibody (mAb 3E9) that specifically recognizes SADS-CoV nucleocapsid (N) protein was used to estimate the progress of infection [36]. The results showed that the amounts of LC3-II increased at 24 and 36 h after SADS-CoV infection, whereas the amounts of LC3-II did not substantially change in the mock-infected Vero E6, ST, and IPI-2I cells (Fig 1B). -These data suggested that autophagosomes accumulated in the SADS-CoV-infected cells.

Another marker of autophagic vesicle formation is the punctate accumulation of LC3, which represents the recruitment of LC3-II to phagophores, the precursors of autophagosomes [34]. Vero E6 cells were transfected with a green fluorescent protein-tagged LC3 plasmid (pGFP-LC3), and followed by an examination by confocal immunofluorescence microscopy. The green fluorescent pattern exhibited a typical punctate morphology after SADS-CoV infection, resembling the pattern of autophagosome-like vesicles, as evidenced by the positive red SADS-CoV antigen (viral N proteins) staining. In contrast, the patterns produced by GFP-LC3 in the mock-infected cells were diffused (Fig 1C). These results indicated that SADS-CoV infection induced the formation of autophagosomes.

A recent study showed that infectious bursal disease virus (IBDV) could induce autophagy through binding to a cellular receptor [37]. UV-inactivated viruses are considered to lose their abilities to replicate in host cells. Thus, to examine whether the active replication of SADS-CoV was required for the induction of autophagy, the western blotting was used to evaluate the amounts of LC3-II in ST cells after inoculation with UV-inactivated viruses (corresponding to a multiplicity of infection (MOI) of 0.1). As shown in Fig 1D, LC3-II expression was similar to that in mock-infected cells and in cells inoculated with UV-inactivated SADS-CoV at 36 hours post infection (hpi). In addition, no N protein synthesis was detected in ST cells inoculated with UV-inactivated SADS-CoV. In contrast, ST cells infected with replication-competent SADS-CoV apparently underwent the conversion of LC3-I to LC3-II, thus suggesting that the active replication of SADS-CoV was essential for the induction of autophagy (Fig 1D).

### SADS-CoV replication is regulated by autophagy

**Fig 2:**
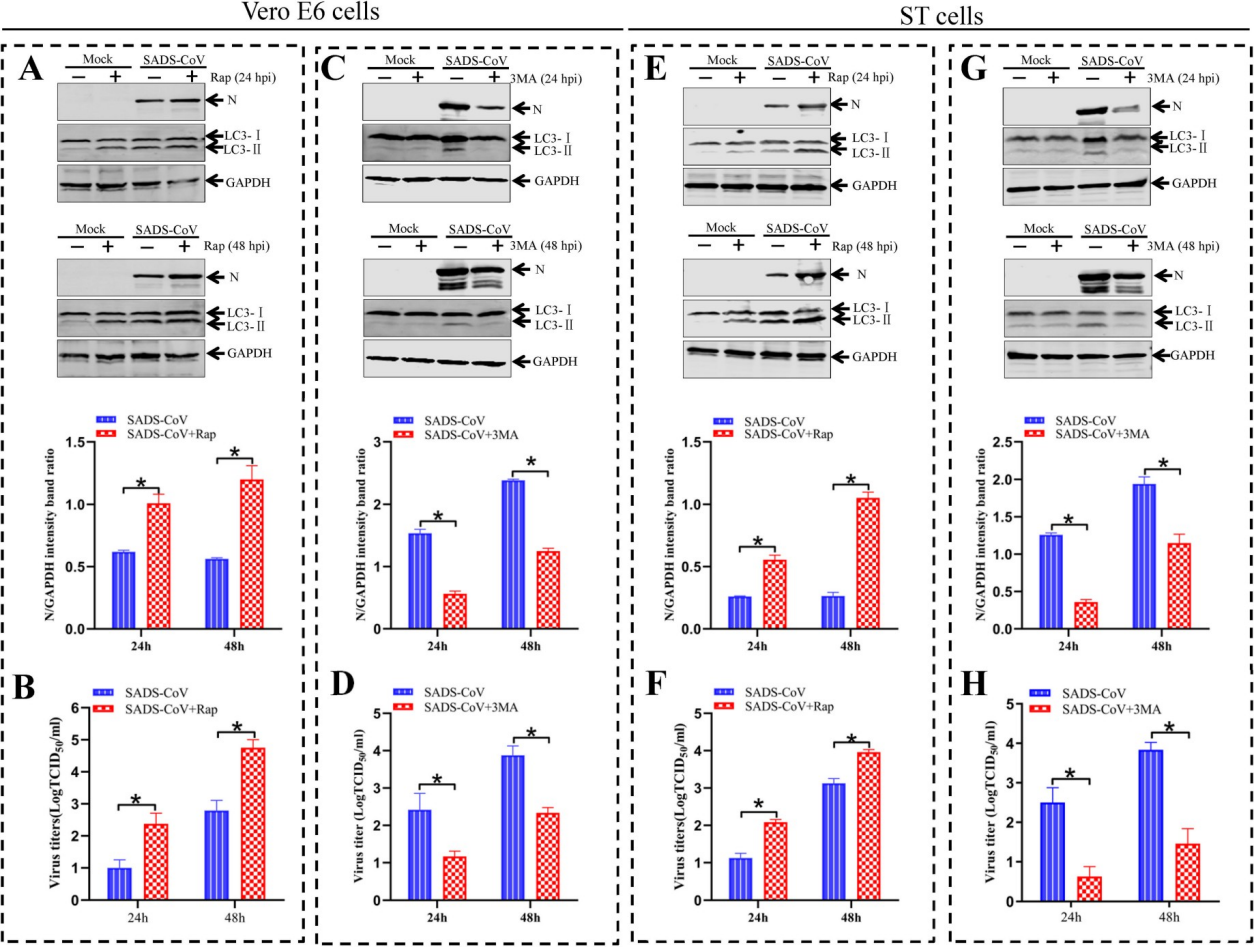
The effect of autophagy on SADS-CoV replication in cells treated with different pharmacological compounds. (**A** and **E**) Vero E6 and ST cells are mock-pretreated or pretreated with complete medium containing 500 nM rapamycin (Rap) for 2 h, and then subjected to SADS-CoV absorption for 1 h, and further cultured in fresh medium in the presence or absence of 500 nM Rap for 24 or 48 h. Cell lysates are analyzed by western blotting using anti-LC3 and -viral N antibodies. The presence of Rap is indicated with “+”. (**B and F**) Vero E6 and ST cells are pretreated and infected as described in A and C. Progeny virus yields are determined based on 50% tissue culture infective dose (TCID_50_) per 1 mL in Vero E6 cells. (**C and G**) Vero E6 or ST cells are mock-pretreated or pretreated with complete medium containing 2 mM 3-methyladenine (3MA) for 2 h and then subjected to SADS-CoV absorption for 1 h and further cultured in fresh medium in the presence or absence of 2 mM 3MA for 24 or 48 h. Cell lysates are analyzed by western blotting. The presence of 3MA is indicated with “+”. (**D and H**) Vero E6 and ST cells are pretreated and infected as described in C and G. Progeny virus yields are determined based on TCID_50_ per 1 mL in Vero E6 cells. Means and SD (error bars) of three independent experiments are indicated (*P < 0.05).

Given that autophagy was induced by SADS-CoV infection, we continued to determine whether SADS-CoV replication could be regulated by the cellular autophagy. Rapamycin (Rap), a widely used inducer for autophagy, and 3-methyladenine (3MA), a widely used inhibitor for autophagy [38], were used to treat SADS-CoV-infected cells for evaluations of viral protein expression and virus production. As shown in Fig 2A and 2E, the conversion of LC3-I to LC3-II in SADS-CoV-infected Vero E6 and ST cells was increased after Rap pretreatment, thus indicating that autophagy was upregulated by Rap in virus-infected cells. It was also observed that the expression of viral N protein was significantly increased after Rap treatment as compared with the untreated cells (Fig 2A and 2E). Viral yields were also higher in Rap-treated cells than that in untreated cells at 24 and 48 hpi (Fig 2B and 2F). In contrast, 3MA treatment significantly inhibited the conversion of LC3-I to LC3-II and reduced the expression levels of viral N protein as compared with that observed in untreated SADS-CoV-infected cells (Fig 2C and 2G). Viral titers were decreased in 3MA-treated cells at 24 and 48 hpi, respectively (Fig 2D and 2H). In addition, the Cell Counting Kit-8 (CCK-8) assay demonstrated that the viability of Vero E6 and ST cells was not obviously affected by the treatments of Rap or 3MA (S2 Fig). These results further confirmed that autophagy played a critical role during SADS-CoV replication.

### Knockdown of endogenous ATG5 or LC3 genes reduces SADS-CoV replication

**Fig 3:**
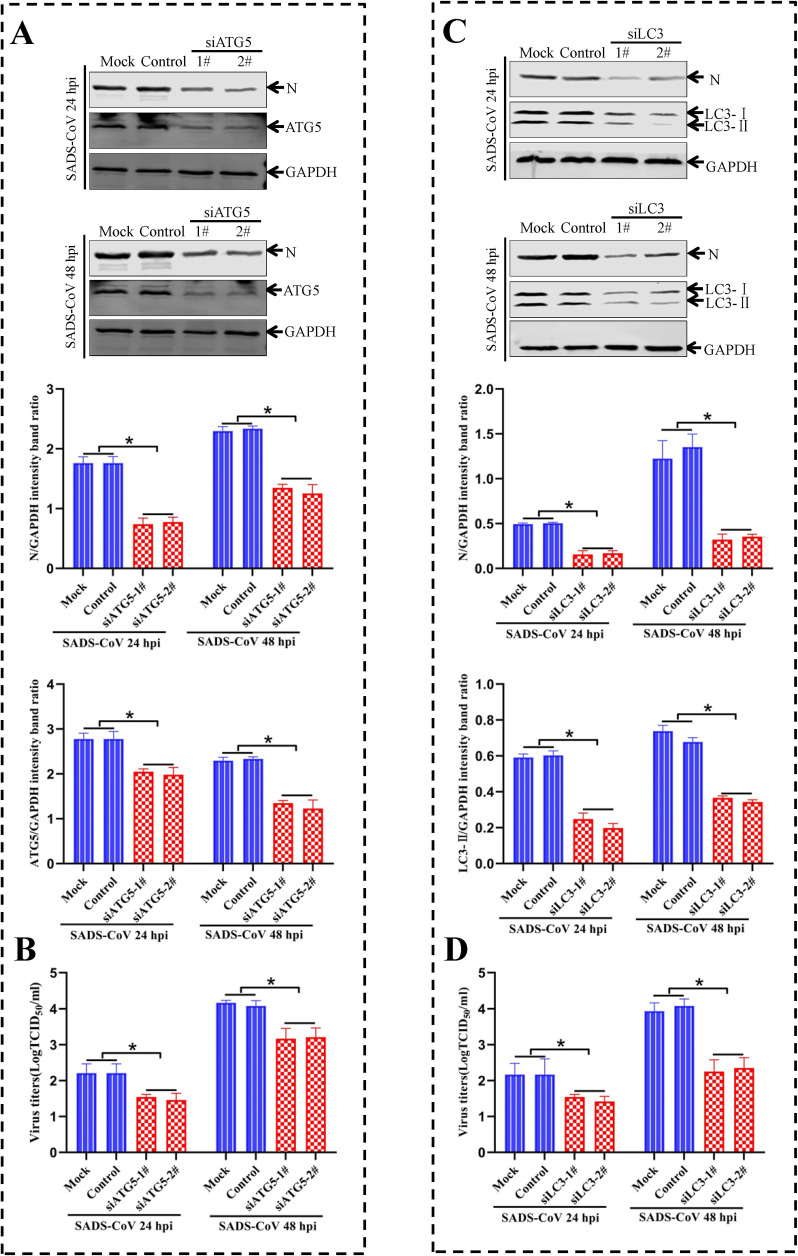
Knockdown of endogenous ATG5 or LC3 genes inhibits the replication of SADS-CoV. (**A and B**) ST cells treated with Mock, Control siRNA (Control) or siATG5 for 48 h, and followed by an infection with SADS-CoV for 24 and 48 h, respectively. The cellular lysates are analyzed by western blotting with anti-viral N, anti-ATG5, or anti-GAPDH antibodies, and the viral titers are determined. (**C and D**) ST cells treated with Mock, Control siRNA (Control), or siLC3 for 48 h, and followed by an infection with SADS-CoV for 24 and 48 h, respectively. The cells are analyzed by western blotting with anti-viral N, anti-LC3, or anti-GAPDH antibodies and the viral titers are determined. Means and SD (error bars) of three independent experiments are indicated (*P < 0.05).

To further explore the relationship between autophagy machinery and viral replication, target-specific RNA interference was employed to down-regulate the intracellular autophagy proteins ATG5 or LC3. The effects on the replication of SADS-CoV were examined. ATG5 is essential for the activation of conventional autophagy and autophagosome formation [39]. In order to confirm a knockdown of autophagic signaling impairing SADS-CoV replication, ST cells were infected with SADS-CoV after transfection with an siRNA targeting to ATG5. Viral loads in the cellular supernatants and N protein levels were used to evaluate the effects of ATG5 knockdown on viral replication. As shown in Fig 3A and 3B, a knockdown of ATG5 in ST cells resulted in a downregulation of N protein expression at 24 and 48 hpi, accompanied by a significant reduction of viral loads in the cellular supernatants in comparison with cells treated with a negative control siRNA (Control), or mock-treated cells (Mock).

As autophagic signaling is induced, increased amounts of the cellular autophagic protein LC3 conjugated to the lipid phosphatidylethanolamine. This conjugation confers membrane association and is required for autophagosome formation and membrane expansion [40]. LC3 was down-regulated with a specific siRNA in ST cells. Viral N protein level was significantly decreased in the LC3 knockdown cells. Likewise, infectious progeny viruses in cellular supernatants were significantly reduced by LC3 siRNA compared with cells treated with a negative control siRNA (Control), or mock-treated cells (Mock) (Fig 3C and 3D). These results indicated that the autophagic vacuole membrane structure was important for viral replication. Taken together, these results further demonstrated that autophagy was required for efficient replication of SADS-CoV.

### Induction of complete autophagy by SADS-CoV is beneficial to viral replication

**Fig 4:**
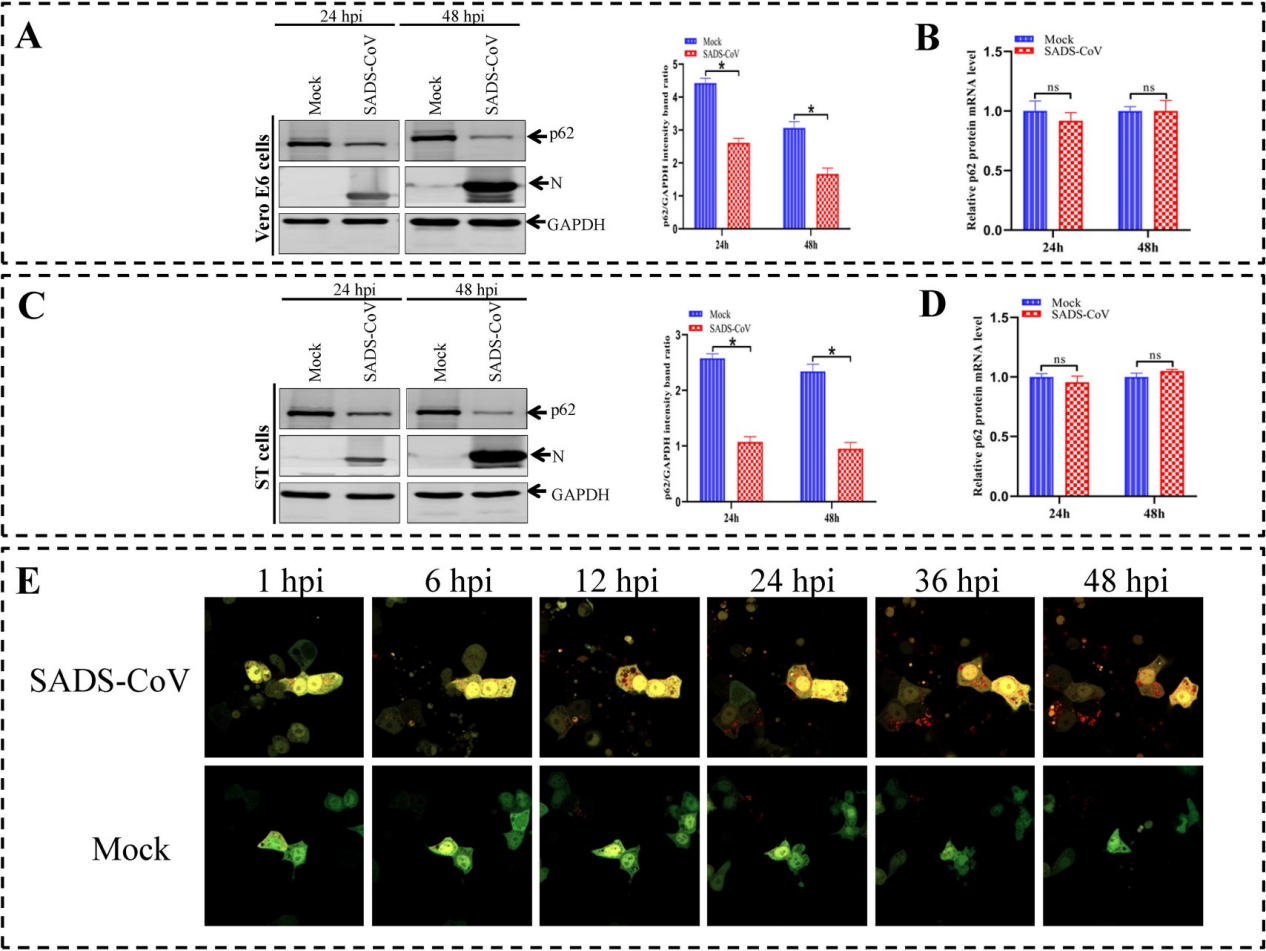
SADS-CoV-induced complete autophagy. (**A and C**) Vero E6 and ST cells are mock-infected or infected with SADS-CoV at the indicated time points (24 and 48 hpi), the cellular lysates are subjected to a western blotting analysis with anti-viral N and anti-p62 antibodies. GAPDH is used as a protein loading control. (B and D) Vero E6 and ST cells are pretreated and infected as described in A and C. Real-time RT-PCR was performed to measure p62 mRNA levels. (**E**) Vero E6 cells are transfected with plasmid GFP-RFP-LC3 for 12 h, and cells were infected or mock-infected with SADS-CoV at an MOI of 1. After absorption for 1 h, cells were observed over time by live cell imaging with RFP and GFP channels. Serial pictures obtained from the video at the indicated time points are shown. Means and SD (error bars) of three independent experiments are indicated (*P < 0.05).

The p62/SQSTM1 protein is a selective autophagic molecule that is incorporated into autophagosomes, and followed by being degraded by lysosomal hydrolases [41]. These events represent a complete autophagic process, which induces autophagic flux and autophagosome maturation. To elucidate whether the autophagy induced by SADS-CoV infection was a complete or incomplete autophagic process, two methods were used: (1) The consumption of the p62 autophagy adaptor was examined in the SADS-CoV-infected Vero E6 and ST cells; (2) A plasmid containing a GFP-RFP tandem fluorescent-tagged LC3 (GFP-RFP-LC3) was used. The fusion protein GFP-LC3 was relatively stable, but the low pH in the autophagosomes quenched the GFP fluorescent signal. In contrast, RFP-LC3 exhibited more stable fluorescence in acidic compartments, making the GFP-RFP-LC3 tandem construct useful for assessing autophagosome dynamics [42]. The expression of p62 in Vero E6 and ST cells was examined after an infection of SADS-CoV at 24 and 48 hpi, respectively. The level of p62 was decreased in the SADS-CoV-infected cells as compared with that in the uninfected cells (Fig 4A and 4C). No alteration of p62 mRNA expression was observed in the SADS-CoV-infected cells as assessed by real-time RT-PCR (Fig 4B and 4D). These results indicated that autophagic flux occurred in the SADS-CoV-infected cells. The GFP-RFP tandem fluorescent-tagged LC3 plasmid (GFP-RFP-LC3) was used to detect autophagic flux by using a live cell imaging system. A large number of red autophagosome vacuoles were observed in SADS-CoV-infected cells, but not in the mock-infected cells (Fig 4E). Subsequently, the yellow puncta turned red, most likely because the quenching of GFP-LC3 fluorescence in the acidic amphisomes or autophagosomes (Fig 4E and S1 Movie). This color change indicated the maturation of autophagosomes. In contrast, the few yellow or red LC3 puncta represented the baseline range of autophagic flux in the mock-treated cells (Fig 4E and S2 Movie).

### Autophagosomes enhance SADS-CoV replication

**Fig 5:**
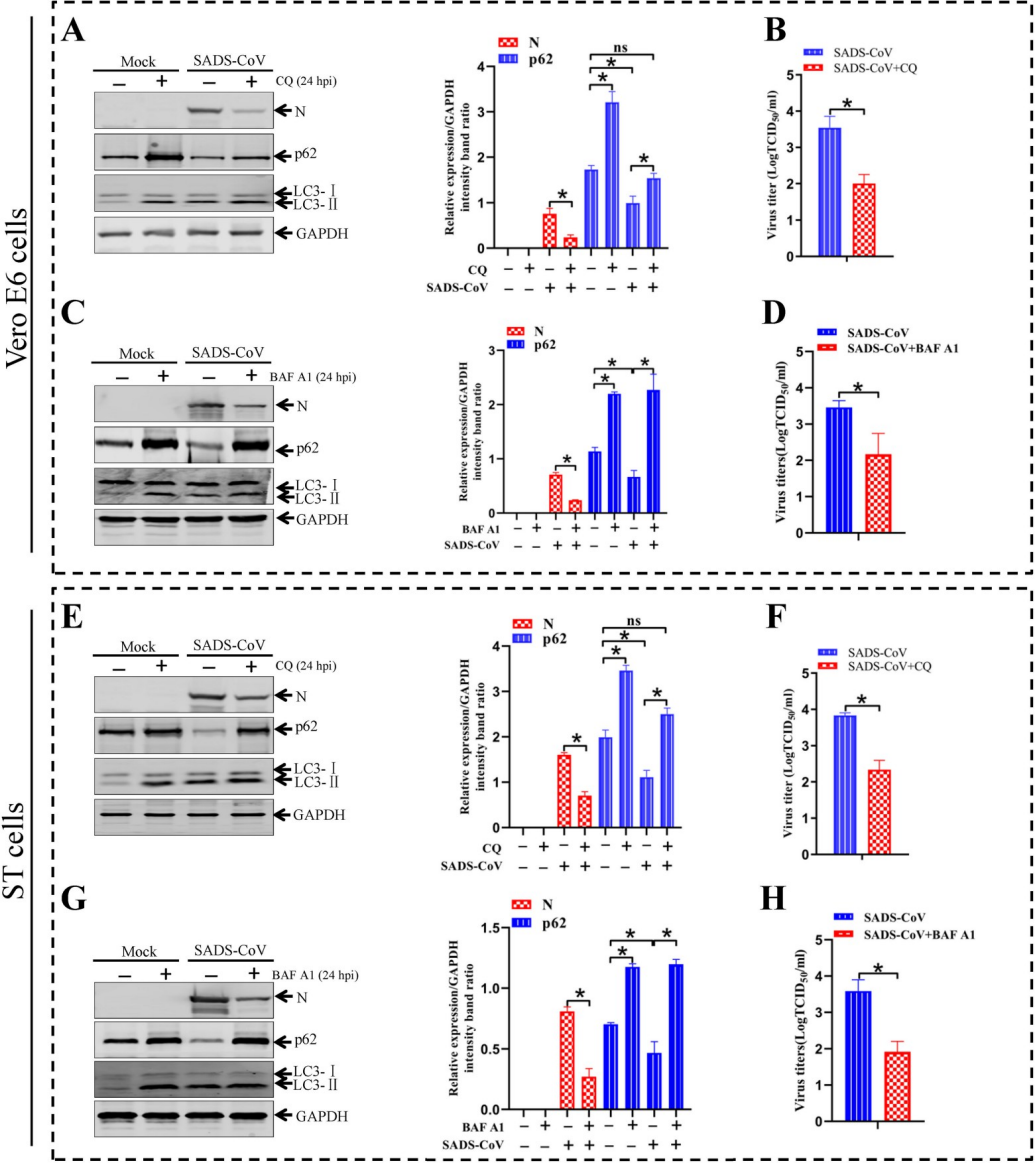
SADS-CoV-induced complete autophagy is beneficial for viral replication. Vero E6 and ST cells are mock-pretreated or pretreated with complete medium containing 20 mM chloroquine (CQ) (A and E) or 20 nM BAF A1 (C and G) for 2 h and then subjected to SADS-CoV absorption for 1 h, and further cultured in fresh medium in the presence or absence of 20 mM CQ or 20 nM BAF A1 for 24 hpi. Cell lysates are analyzed by western blotting using anti-viral N, anti-p62, anti-LC3, and anti-GAPDH antibodies. The presence of CQ or BAF A1 is indicated with “+”. (**B, D, F and H**) Vero E6 and ST cells are pretreated and infected as described above. The titers of progeny virus yields are determined with TCID_50_ at 24 hpi in Vero E6 cells. Means and SD (error bars) of three independent experiments are indicated (*P < 0.05).

Cargo is hydrolyzed in acidic lysosomes. Chloroquine (CQ) can inhibit lysosome acidification, and thereby inhibiting the hydrolysis activity of lysosomes [43]. In order to elucidate whether SADS-CoV replication can be influenced by the loss of lysosomal hydrolysis activity in the presence of CQ, SADS-CoV-infected Vero E6 or ST cells were treated with CQ. As shown in Fig 5A and 5E, CQ treatment resulted in an increase in both LC3-II and p62 levels. Cells treated with CQ showed an increase in both LC3-II and p62, indicating that autophagic signaling was induced and hydrolysis by lysosomes was inhibited. Moreover, viral N protein expression was significantly decreased after a treatment of CQ (Fig 5A and 5E). The viral titers in CQ-treated cells were significantly decreased as compared with that in the SADS-CoV-infected cells at 24 hpi (Fig 5B and 5F). Bafilomycin A1 (BAF A1) is widely used as an inhibitor of autophagosome-lysosome fusion *in vitro* to determine the activity of autophagic flux on viral replication. As shown in Fig 5C and 5G, BAF A1 treatment resulted in inhibition of autophagosome fusion with lysosomes, which increased levels of LC3-II and p62. Cells treated with BAF A1 showed that autophagic signaling was induced but that fusion of autophagosomes with lysosomes was inhibited with an increase in both LC3-II and p62. As shown in Fig 5C and 5G, viral protein N showed a significant decrease in the presence of BAF A1. Inhibition of autophagosome fusion significantly reduced the infectious progeny viral titers (Fig 5D and 5H). Taken together, these results demonstrated that SADS-CoV infection enhanced autophagosome maturation and induced a complete autophagic process, which was beneficial to viral production.

### SADS-CoV infection activates all three UPR pathways *in vitro* and *in vivo*, and ER stress contributes to SADS-CoV-induced autophagy

**Fig 6:**
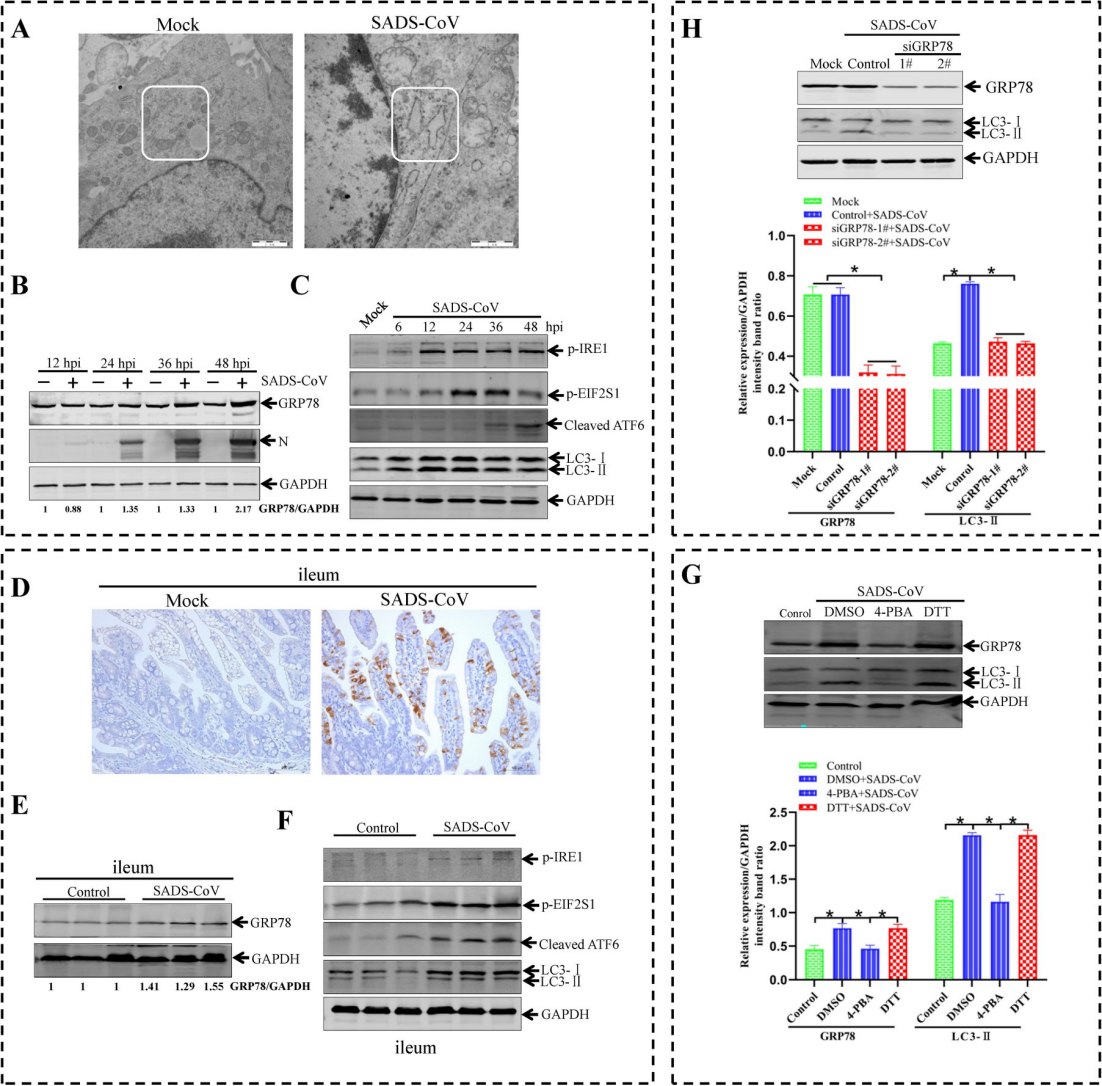
SADS-CoV triggers autophagy through activating ER stress. (**A-C**) SADS-CoV infection causes ER stress by activating all three UPR pathways *in vitro*. (**A**) Expansion of the ER is detected with transmission electron microscopy (TEM). Vero E6 cells, either mock-infection or infection with SADS at an MOI of 0.1, are analyzed for the ER expansion via electron microscopy at 36 hpi. The square region highlights ER stress. Scale bar indicates 1 μm. (**B**) ST cells are mock mock-infected or infected with SADS-CoV at the indicated time points (12, 24, 36 and 48 hpi) to analyze GRP78 protein expression by western blotting using anti-viral N, anti-GRP78, and anti-GAPDH antibodies. The densities of GRP78 bands were analyzed to calculate the values relative to that of GAPDH. Results were normalized to respective control cells. (**C**) SADS-CoV infection activates all three UPR signaling pathways *in vitro*. ST cells are infected with SADS-CoV at an MOI of 0.1 and harvested at 6, 12, 24, 36, and 48 hpi. Cell lysates are subjected to a western blotting analysis with antibodies against p-IRE1, p-EIF2S1, ATF6, and LC3. GAPDH is used as a loading control. (**D-F**) SADS-CoV infection causes ER stress by activating all three UPR signaling pathways *in vivo*. (**D**) Representative microphotographs of viral antigen immunochemical staining in SADS-CoV-noninfected and -infected ileum tissue. Scale bars indicate 100 μm. (**E and F**) Protein levels of GRP78, p-IRE1, p-EIF2S1, cleaved ATF6, and LC3 in ileum samples of SADS-CoV-noninfected and -infected piglets. The densities of GRP78 bands were analyzed to calculate the values relative to that of GAPDH. Results were normalized to respective control piglet. (**H**) ST cells are transfected with the siRNAs of GRP78 or siRNAs of negative control for 48 h, and followed by an infection with SADS-CoV at an MOI of 0.1 or mock infection. After 1 h of virus absorption at 37°C, the cells are further cultured in maintenance medium. At 36 h after infection with mock or SADS-CoV, the cells are subjected to western blotting using anti-GRP78, anti-LC3 and anti-GAPDH antibodies. (**G**) Western blotting is used to analyze the expression of GRP78 and LC3 in SADS-CoV-noninfected and -infected cells in the absence or presence of the ER stress inhibitor 4-PBA (1 mM) or the ER stress inducer DTT (1 mM). Means and SD (error bars) of three independent experiments are indicated (*P < 0.05).

ER is an organelle in eukaryotic cells serving for several critical functions, such as protein synthesis folding, modification, and trafficking [20,29]. When proteins entering the ER overwhelm its folding capacity, misfolded/unfolded proteins accumulate in the ER and cause ER stress. It was reported that autophagy was induced via the ER stress response [44]. To elucidate how SADS-CoV infection induced autophagy, we investigated whether ER stress was present and UPR pathways were activated in the SADS-CoV-infected ST cells. First, TEM was used to observe the ER lumen in the SADS-CoV-infected cells. A greatly expanded ER lumen (a morphological marker of ER stress) could be observed in SADS-CoV-infected cells as compared with mock-infected cells (Fig 6A). Moreover, GRP78, a biomarker of ER stress, was significantly increased in the SADS-CoV-infected ST cells relative to mock-infected cells (Fig 6B). Three ER transmembrane-proximal sensors that act as UPR transducers are IRE1, ATF6, and PERK [45]. To determine whether SADS-induced autophagy can be attributed to ER stress-mediated UPR signaling, the activation of the three UPR pathways was examined during SADS-CoV infection *in vitro*. As shown in Fig 6C, IRE1 and EIF2S1 were phosphorylated, and cleaved ATF6 (50-kDa protein) was increased during SADS-CoV infection in ST cells. These data suggested that SADS-CoV infection triggered all three pathways *in vitro*. To explore whether SADS-CoV infection could induce ER stress *in vivo*, ileum tissue from SADS-CoV-infected piglets at 36 hpi was dissected. SADS-CoV infection was confirmed by immunohistochemistry with a specific mAb (3E9) against the SADS-CoV N protein (Fig 6D) [36]. GRP78 level was increased in SADS-CoV-infected ileum tissue as compared with the control (Fig 6E). These results demonstrated that SADS-CoV infection induced cellular ER stress *in vivo* as well as *in vitro*. To further determine if all three UPR branches were activated *in vivo*, the UPR signaling pathways in SADS-CoV-infected piglets were examined. The phosphorylation of IRE1, EIF2S1, and the cleaved ATF6 was increased in SADS-CoV-infected ileum tissue as compared with controls (Fig 6F). The results also showed that the amounts of LC3-II increased after SADS-CoV infection, whereas the amounts of LC3-II were not substantially changed in the mock-infected piglets. These data demonstrated that SADS-CoV infection activated autophagy and all three UPR pathways *in vivo*.

To confirm if SADS-CoV-triggered ER stress was related to SADS-CoV-induced autophagy, siRNAs were used to down-regulated endogenous GRP78 that could block ER stress during the infection progress. As shown in Fig 6H, blocking ER stress using siGRP78 significantly suppressed the conversion of LC3-I to LC3-II in the SADS-CoV-infected ST cells compared to that in control siRNA and mock-treated cells. In addition, the conversion of LC3-I to LC3-II was also suppressed in SADS-CoV-infected cells treated with 4-phenylbutyric acid (4-PBA, an inhibitor of ER stress) (Fig 6G). Thus, SADS-CoV-induced autophagy could be inhibited by blocking ER stress. In contrast, pretreatment of cells with DL-dithiothreitol (DTT, an inducer of ER stress) promoted the conversion of LC3-I to LC3-II. These results indicated that SADS-CoV infection could trigger ER stress, providing insight into the mechanism of autophagy induction.

### The ER stress sensor IRE1, but not PERK-EIF2S1 and ATF6, is involved in SADS-CoV-induced autophagy and facilitates SADS-CoV replication

**Fig 7:**
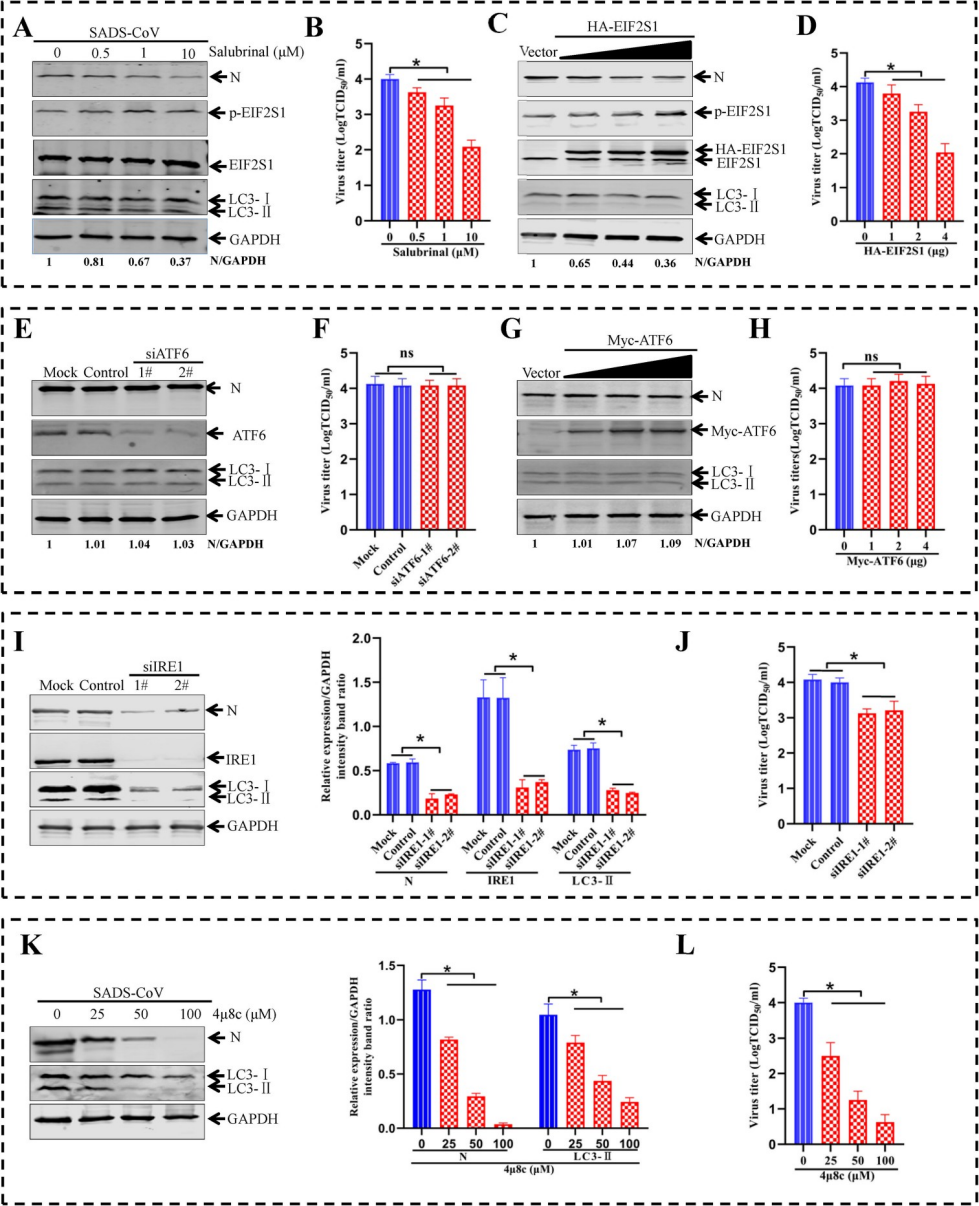
The ER stress sensor IRE1, but not PERK-EIF2S1 and ATF6, is involved in SADS-CoV-induced autophagy and facilitates SADS-CoV replication. (**A and B**) ST cells are treated with the indicated concentrations of salubrinal for 2 h and then infected with SADS-CoV (MOI = 0.1). After the adsorption of viruses to cells, media containing the indicated concentrations of salubrinal is added. Cellular lysates of ST cells are analyzed by western blotting using antibodies against SADS-CoV N protein, p-EIF2S1, EIF2S1, LC3, or GAPDH. The densities of N bands were analyzed to calculate the values relative to that of GAPDH. Results were normalized to group 0 μm of salubrinal, which were set as 1. The titers of SADS-CoV in the supernatants are determined on Vero E6 cells. (**C and D**) ST cells are transfected with pCAGGS-HA (Vector) or pHA-EIF2S1 for 24 h and then infected with SADS-CoV. At 36 hpi, western blotting is performed to detect levels of SADS-CoV N, p-EIF2S1, EIF2S1, LC3 or GAPDH, and the viral titers are determined. The densities of N bands were analyzed to calculate the values relative to that of GAPDH. Results were normalized to group Vector, which were set as 1. (**E and F**) ST cells treated with Mock, Control siRNA or siATF6 for 48 h are infected with SADS-CoV for 36 h. Western blotting is performed to detect levels of SADS-CoV N, ATF6, LC3, or GAPDH, and the viral titers are determined. The densities of N bands were analyzed to calculate the values relative to that of GAPDH. Results were normalized to mock cells, which were set as 1. (G **and H**) ST cells are transfected with pCMV-Myc (Vector) or pCMV-Myc-ATF6 for 24 h and then infected with SADS-CoV. At 36 hpi, western blotting is performed to detect levels of SADS-CoV N, Myc, LC3 or GAPDH, and the viral titers are determined. The densities of N bands were analyzed to calculate the values relative to that of GAPDH. Results were normalized to group Vector, which were set as 1. (**I and J**) ST cells treated with Mock, Control siRNA or siIRE1 for 48 h are infected with SADS-CoV for 36 h. Western blotting is performed to detect levels of SADS-CoV N, IRE1, LC3, or GAPDH, and the viral titers are determined. (**K and L**) ST cells are treated with the indicated concentrations of 4μ8C for 2 h and then infected with SADS-CoV (MOI = 0.1) in the presence of 4μ8C. (**K**) After the adsorption of viruses to cells, media containing the indicated concentrations of 4μ8C is added. Cell lysates of ST cells are analyzed by western blotting using antibodies against SADS-CoV N protein, LC3, or GAPDH. (**L**) The titers of SADS-CoV in the supernatants is determined on Vero E6 cells. Means and SD (error bars) of three independent experiments are indicated (*P < 0.05).

SADS-CoV infection triggered three UPR signaling pathways (IRE1, ATF6, and PERK-EIF2S1) (Fig 6C and 6F). Next, it was important to determine the UPR pathway that primarily accounted for the accumulation of LC3-II during SADS-CoV replication. To verify the role of PERK-EIF2S1 pathway during SADS-CoV replication and accumulation of LC3-II, PERK-EIF2S1 signaling pathway was disrupted with a EIF2S1-specific inducer salubrinal (a selective inhibitor of EIF2S1 dephosphorylation). Salubrinal treatment increased the level of phosphorylated EIF2S1 in the SADS-CoV-infected cells (Fig 7A). Meanwhile, the increase in phosphorylated EIF2S1 by salubrinal treatment caused a decrease of SADS-CoV N protein expression and viral titers in the supernatants (Fig 7A and 7B). Furthermore, ST cells were transiently transfected with plasmid expressing HA-EIF2S1, and then infected with SADS-CoV for 36 h. The level of SADS-CoV N protein was reduced when p-EIF2S1 was upregulated (Fig 7C). A reduction of viral titers was observed in the cellular supernatants (Fig 7D). However, the accumulation of LC3-II was not changed in the cells after salubrinal treatment and EIF2S1 overexpression (Fig 7A and 7C). These data demonstrated that the PERK-EIF2S1 signaling pathway may negatively regulate SADS-CoV infection, but further study is needed to confirm this phenomenon.

To investigate the effect of ATF6 pathway on SADS-CoV replication and accumulation of LC3-II, the level of ATF6 was either decreased by a specific siRNA or increased by transfection with a recombinant plasmid expressing Myc-ATF6. First, a specific siRNA was used to silence the ATF6 gene in ST cells. No significant difference was observed in SADS-CoV N protein expression and viral titers after ATF6 knockdown as compared to that in control siRNA (Control) treated cells, or mock-infected cells (Mock) (Fig 7E and 7F). Subsequently, the accumulation of LC3-II was not different between ATF6 knockdown cells and controls (Fig 7E). Next, the ST cells were transfected with plasmid pCMV-Myc-ATF6 and then infected with SADS-CoV. As shown in Fig 7G, the accumulation of LC3-II or N protein expression was not significantly changed in the pCMV-Myc-ATF6-transfected cells as compared with pCMV-Myc plasmid-transfected ones. Moreover, difference of viral yields was not observed between the ATF6 overexpression group and the control group (Fig 7H). Taken together, these results indicated that the activation of PERK-EIF2S1 and ATF6 branches did not account for the facilitation of SADS-CoV replication by induced autophagy.

ER stress has been shown to induce cyto-protective autophagy through the IRE1 pathway [46]. Therefore, the involvement of IRE1 pathway in SADS-CoV-induced autophagy and viral replication was examined. Toward this end, the IRE1 was downregulated by using RNAi. The knockdown of IRE1 decreased SADS-CoV N protein level and accumulation of LC3-II (Fig 7I), accompanied by a significant reduction of viral loads in cell culture supernatants in comparison with that in control siRNA treated cells and mock-treated cells(Fig 7J). These findings are consistent with the effects of an IRE1-specific inhibitor (4μ8C) [47] on SADS-CoV N protein expression, accumulation of LC3-II, and viral replication (Fig 7K and 7L). Thus, these data indicated that IRE1 enhanced SADS-CoV replication by autophagy.

### The membrane-associated papain-like protease PLP2 of SADS-CoV acts as an autophagy-inducing protein

**Fig 8:**
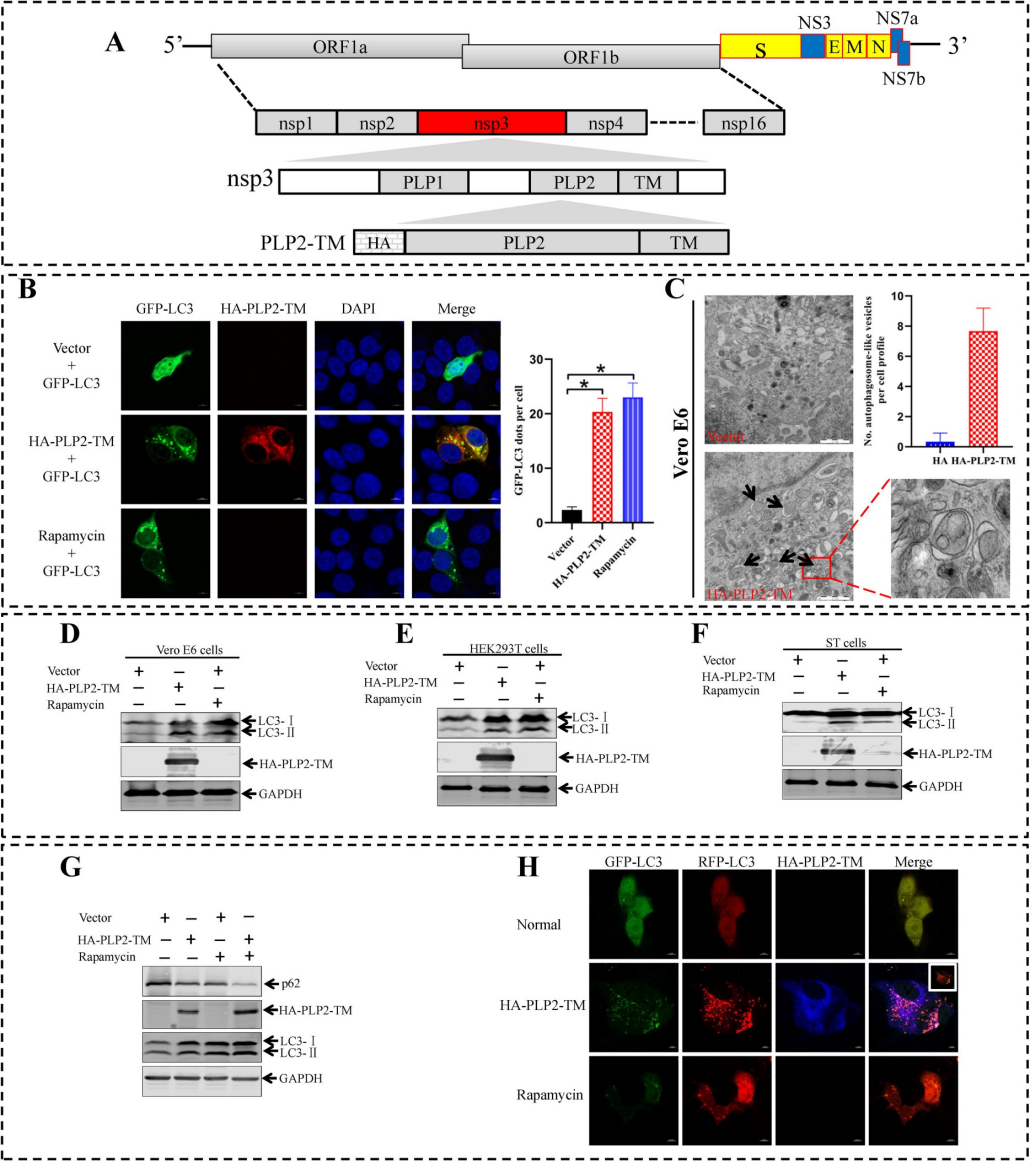
PLP2-TM is a novel autophagy-inducing protein encoded by SADS-CoV. (**A**) Schematic diagram illustrates the coronavirus SADS-CoV genome, polyprotein (pp) 1a/b and the predicted processing of replicase polyproteins (pp) to nsps. The domains, including the predicted transmembrane (TM) domain in nsp3 and the membrane anchored-PLP2 construct (PLP2-TM) used in this study are indicated. (**B**) The HA-PLP2-TM plasmid is co-transfected with pGFP-LC3 into Vero E6 cells. As a positive control for the induction of autophagy, Vero E6 cells are transfected with GFP-LC3 for 24 h and then treated with complete medium supplemented with 250 nM rapamycin for 6 h. The immunofluorescence of cells is detected with confocal microscopy after staining with anti-HA-tagged primary antibody and then with Alexa Fluor 594-conjugated goat anti-rabbit secondary antibody. The localization of GFP-LC3 positive autophagosome accumulation (green) and the HA-tagged PLP-TM (anti-HA, red) are visualized with confocal microscopy. (**C**) SADS-CoV PLP2-TM induces autophagosome-like structures detected with transmission electron microscopy. Vero E6 cells are transfected with pCAGGS-HA or HA-PLP2-TM for 36 h, and then analyzed for autophagosome formation with transmission electron microscopy. Black arrows indicate representative autophagosome-like structures. Scale bar indicates 1 μm. (**D-F**) Vero E6, HEK293T and ST cells are transfected with HA-PLP2-TM or empty vector for 36 h. The cells are treated with 250 nM rapamycin to serve as a positive control for the induction of autophagy. The expression levels for each protein are analyzed by western blotting analysis with the indicated antibodies (Right panel). (**G**) Vero E6 cells are transfected with pCAGGS-HA or HA-PLP2-TM. As a positive control for the induction of autophagy, Vero E6 cells are treated with complete medium supplemented with 250 nM rapamycin for 6 h. At 36 h post-transfection, the levels of endogenous autophagic substrate p62, LC3, and PLP2-TM are determined with western blotting. GAPDH is used as a protein loading control. (**H**) PLP2-TM activates autophagosome formation and fusion with lysosomes. Vero E6 cells are co-transfected with GFP-RFP-LC3 and HA-PLP2-TM. As a positive control for the induction of autophagy, Vero E6 cells are transfected with GFP-RFP-LC3 and then treated with complete medium supplemented with 250 nM rapamycin for 6 h. On the basis of differential pH sensitivities for RFP and GFP, the GFP-RFP-LC3 displays red and green in nonacidified autophagosomes (red^+^green^+^, yellow in merged images), and red in acidified, degradative autolysosomes (red^+^green^−^, red in merged images). Means and SD (error bars) of three independent experiments are indicated (*P < 0.05).

The PLP2-TM of HCoV-NL63 and PEDV, and PLpro-TM of SARS-CoV-1 and MERS-CoV, have been shown to represent a novel class of viral proteins encoded by coronaviruses that induce autophagy [48]. To determine whether SADS-CoV PLP2-TM could regulate autophagy, we first investigated the effects of ectopic expression of SADS-CoV PLP2-TM on the formation of autophagosomes (Fig 8A). Vero E6 cells were co-transfected with plasmids expressing GFP-LC3 and HA-PLP2-TM. The cells were subjected to an examination of autophagosome formation by confocal microscopy at 24 h post-transfection. The cells treated with rapamycin, a well-known autophagy inducer, for 6 h after a transfection with GFP-LC3 for 24 h, were used as a positive control. As shown in Fig 8B, the green fluorescent pattern exhibited obvious puncta of autophagosome-like vesicles in cells with ectopic expression of PLP2-TM and in cells with short-term treatment with rapamycin. In contrast, the patterns produced by GFP-LC3 in the mock-transfected cells were diffused. These results suggested that the PLP2-TM encoded by SADS-CoV was a novel autophagy inducer. To corroborate this finding, we performed TEM to directly visualize autophagosomes in cells expressing PLP2-TM, and the cells transfected with the empty vector were used as a negative control. Double membrane vacuoles containing cytoplasmic organelles, which is a characteristic of autophagosomes, were readily visible in the cytoplasm of PLP2-TM-transfected cells. In contrast, no autophagosome-like structures were observed in cells transfected with the empty vector. The number of autophagic vacuoles per cell was significantly higher in the PLP2-TM-transfected cells than that in the empty vector-transfected cells (Fig 8C). These data provided morphological evidence that PLP2-TM promoted autophagosome accumulation. Furthermore, the expression levels of endogenous LC3-I and LC3-II were examined in cells after an overexpression of PLP2-TM, in comparison with cells transfected with the control vector. In agreement with the GFP-LC3 fluorescence and TEM data, ectopic expression of PLP2-TM substantially increased the abundance of LC3-II in Vero E6, HEK293T, and ST cells (Fig 8D–8F), however, the nsp4 shares a number of features like the presence of multiple membrane-spanning domains with PLP2-TM, but does not induce the accumulation of LC3-II (S1 Fig). Collectively, these data confirmed that an induction of autophagy by PLP2-TM was not a cell-type specific phenomenon. The terminal step in autophagy is the fusion of autophagosomes with lysosomes to form autolysosomes and the subsequent degradation of the contents. We wondered whether the PLP2-TM-induced accumulation of autophagosomes might result in enhanced autophagic degradation. Therefore, the immunoblotting was used to determine the protein levels of a well-characterized autophagic substrate, p62, which combines LC3 and is specifically degraded as a result of complete autophagic flux, in PLP2-TM-expressing Vero E6 cells. As shown in Fig 8G, LC3-II accumulation was increased, and a significant decrease in p62 was observed in the PLP2-TM-transfected Vero E6 cells as compared with mock-infected cells. Rapamycin, which induced complete autophagy, not only increased the LC3-II levels but also caused a significant decline in p62 expression. Moreover, the decreased p62 expression induced by rapamycin was augmented by PLP2-TM. A tandem-tagged fluorescent reporter, GFP-RFP-LC3, was used to clarify the mechanism underlying PLP2-TM induced complete autophagy. In PLP2-TM expressing or rapamycin treated cells, using GFP-RFP-LC3 as an indicator, complete autophagic flux was observed, exhibiting predominant red fluorescence with only dim green fluorescence (Fig 8H). In contrast, the cells co-expressing empty vector and GFP-RFP-LC3 displayed a strong co-localization of red and green fluorescence (shown as yellow in the merged macrograph). These data revealed that PLP2-TM induced complete autophagy, and autophagosome accumulation and autophagic cargo hydrolysis were connected.

### The interaction of PLP2-TM and GRP78 activates the IRE1-JNK-Beclin 1 pathway and induces autophagy

**Fig 9:**
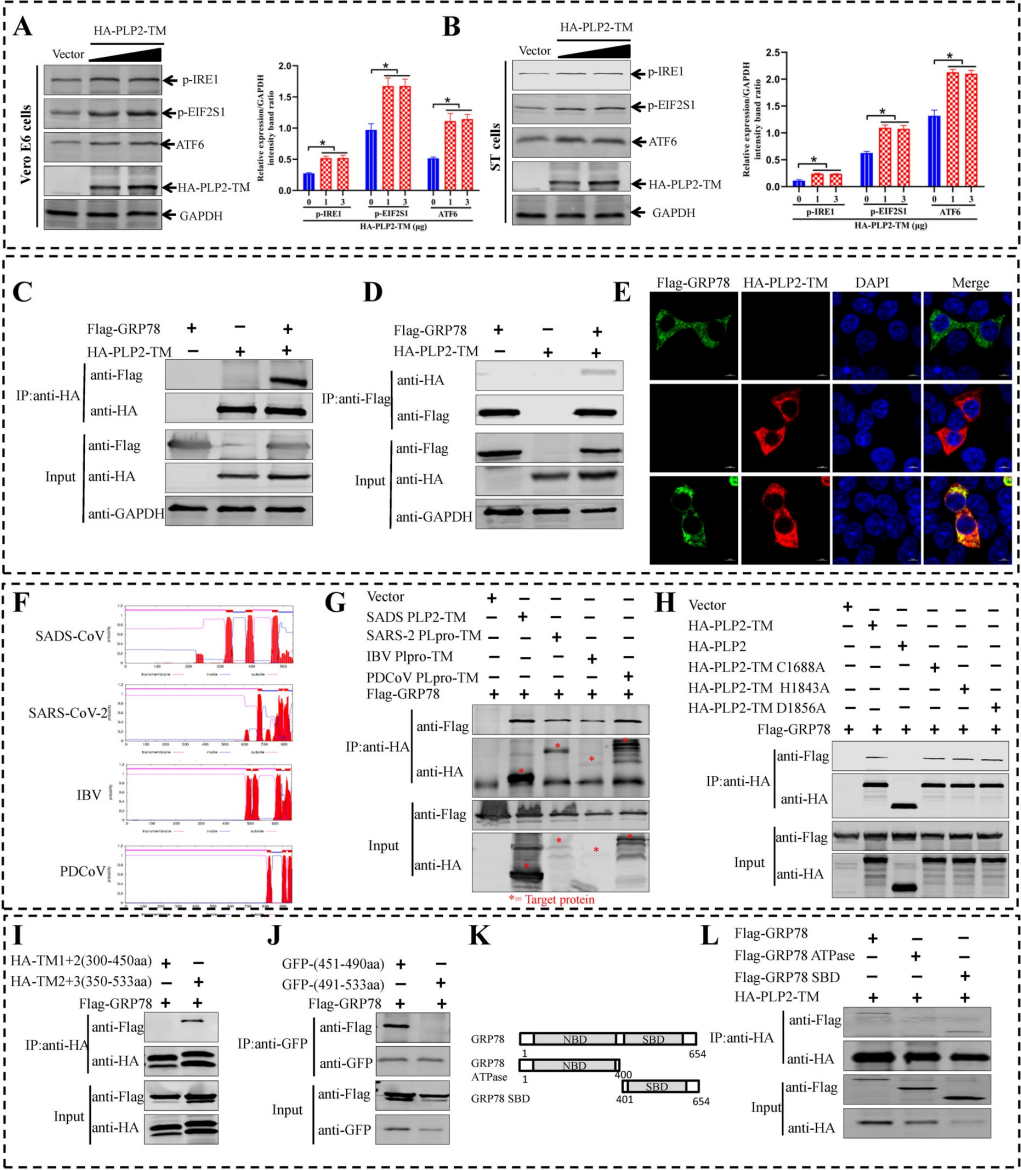
SADS-CoV PLP2-TM activates all three UPR signaling pathways and interaction with GRP78 protein. (**A and B**) Vero E6 and ST cells are transfected with increasing doses of pCAGGS-HA-PLP2-TM. At 36 hpi, western blotting is performed using the indicated antibodies (left panel). Levels of p-IRE1, p-EIF2S1 and cleaved ATF6 relative to the level of GAPDH are determined by a densitometric analysis. Means and SD (error bars) of three independent experiments are indicated (*P < 0.05). (**C and D**) Co-IP analysis of the interaction between SADS-CoV PLP2-TM protein and GRP78 in HEK293T cells. (**E**) Co-localization of PLP2-TM and GRP78. Vero E6 cells are co-transfected with plasmids expressing HA-PLP2-TM and Flag-GRP78. The PLP2-TM is colored green, and the GRP78 fusion protein is colored red. Merged images are also presented, and the position of the nucleus is indicated by DAPI (blue) staining in the merged images. (**F**) The transmembrane motifs in SADS-CoV (*Alphacoronavirus*), SARS-CoV-2 (*Betacoroavirus*), IBV (*Gammacoronavirus*), and PDCoV (*Deltacoronavirus*) nsp3 proteins are predicted with the online tool TMHMM server, version 2.0. (**G** and **H**) Co-IP analysis of the interaction between PLPs and GRP78 derived from various coronavirues. The interaction of SADS-CoV PLP2-TM and GRP78 is independent on the protease activity. (**I** and **J**) The domain of PLP2-TM serving for an interaction with GRP78 is identified. (**K**) Schematics of GRP78 truncations. (**L**) The domain of GRP78 serving for an interaction with PLP2-TM is identified.

Due to UPR was induced by SADS-CoV infection *in vivo* and *in vitro*, we continued to examine whether PLP2-TM protein played a role during this progress. The PLP2-TM protein was heterogeneously expressed in Vero E6 and ST cells. Interestingly, the results were similar to those for SADS-CoV infection, all three pathways of the UPR were activated. However, PLP2-TM treatment dose independently up-regulated the phosphorylation of IRE1, EIF2S1, and cleaved ATF6 (a 50-kDa protein) (Fig 9A and 9B). GRP78 is the master regulator of the UPR, and mediates the UPR through its interactions with three ER resident proteins, PERK, ATF6, and IRE1. Under non-stress conditions, GRP78 binds to PERK, ATF6 and IRE1, whereas these molecules are released from GRP78 and subsequently mediate the UPR by down-regulating protein translation under stress conditions, thus increasing protein degradation and up-regulating the chaperone ability of the ER [49]. Therefore, it was of great interest to detect the interaction of PLP2-TM and GRP78. To explore this interaction, the HA-PLP2-TM and Flag-GRP78 plasmids were co-transfected into HEK-293T cells, and co-immunoprecipitation (Co-IP) was performed using anti-HA and anti-Flag antibodies. The results indicated that GRP78 protein was present in the immunoprecipitated complexes containing PLP2-TM (Fig 9C), and PLP2-TM was detected in the immunoprecipitated complexes containing GRP78 (Fig 9D). Additionally, the co-localization of PLP2-TM and GRP78 was verified in Vero E6 cells by indirect immunofluorescent confocal laser microscopy (Fig 9E). SADS-CoV belongs to the *Coronaviridae* family, which is divided into four major genera: *Alphacoronavirus*, *Betacoroavirus*, *Gammacoronavirus*, and *Deltacoronavirus*. The viral PLP2-TM domain is present in the nsp3 of coronaviruses and participates in the proteolytic processing of the N-terminal region of the polyproteins. The nsp3 in SADS-CoV, SARS-CoV-2, IBV, and PDCoV has a similar transmembrane structure (Fig 9F). The interaction of SADS-CoV PLP2-TM and GRP78 prompted us to investigate whether the PLP2-TM homologues encoded by other coronaviruses also have a similar function. Co-IP analyses showed that HA-tagged PLP2-TM or Plpro-TM of coronavirus formed a complex with Flag-tagged GRP78 (Fig 9G). To determine the specific region in PLP2-TM of SADS-CoV that responsible for the interaction with GRP78, a series of HA or GFP-tagged PLP2-TM deletions were used to map the GRP78 binding domain on PLP2-TM protein. Co-IP result indicated that the F451-L490 aa of PLP2-TM was essential for the interaction with GRP78 protein and the catalytic activity was not required for the interaction of GRP78 and PLP2-TM (Fig 9H–9J). Then, the Flag-tagged N- and C-terminal truncation mutants of GRP78 were constructed (Fig 9K). These mutants were co-expressed with HA-PLP2-TM in HEK-293T cells to map the interaction domain on GRP78 by immunoprecipitation assay. The results showed that the C-terminal substrate-binding domain (SBD) of GRP78 was crucial for the interaction with PLP2-TM (Fig 9L). Collectively, these results indicated that the interaction of GRP78 and PLP2-TM was common in coronaviruses. In subsequent investigations, efforts are still needed to delineate the molecular details underlying the interplay of coronaviral PLP and the autophagy pathway.

**Fig 10:**
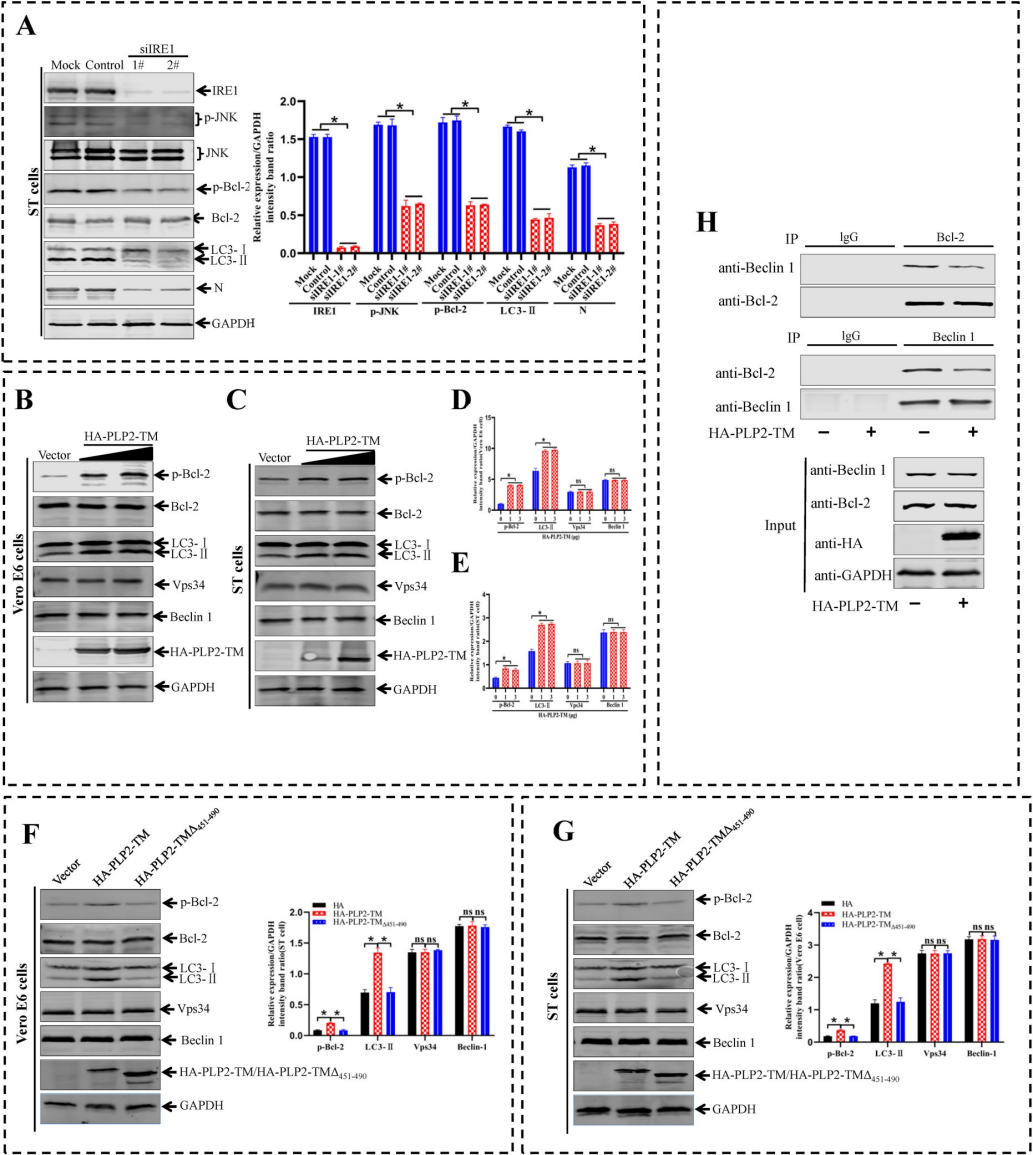
The IRE1-JNK-Beclin 1 signaling pathway is involved in SADS-CoV-induced autophagy. (**A**) ST cells that untreated or treated with Control siRNA or siIRE1 for 48 h, are infected with SADS-CoV for 36 h. A western blotling analysis is performed with the indicated antibodies. Levels of IRE1, p-JNK, p-Bcl-2, LC3-II and SADS-CoV N relative to the level of GAPDH are determined by a densitometric analysis. (**B-E**) Vero E6 (**B and D**) and ST (**C and E**) cells are transfected with increasing doses of pCAGGS-HA-PLP2-TM. At 36 hpi, western blotting is performed with the indicated antibodies. Levels of p-Bcl-2, LC3-II, Beclin 1, and Vps34 relative to the level of GAPDH are determined by a densitometric analysis. (**F and G**) Vero E6 (F) and ST (G) cells are transfected with Vector, HA-PLP2-TM or HA-PLP2-TM_Δ451–490._ At 36 h post-transfection, western blotting is performed with the indicated antibodies. Levels of p-Bcl-2, LC3-II, Beclin 1, and Vps34 relative to the level of GAPDH are determined by a densitometric analysis. (**H**) HEK293T cells are transfected with pCAGGS-HA-PLP2-TM or empty vector. At 36 h post-transfection, cell lysates are subjected to immunoprecipitation with anti-Bcl-2 or anti-Beclin 1 antibodies, and subsequent immunoblotting with anti-Beclin 1 or anti-Bcl-2 antibodies. Means and SD (error bars) of three independent experiments are indicated (*P < 0.05).

The above findings indicated that SADS-CoV induced ER stress and the IRE1 signaling pathway was essential during SADS-CoV-induced autophagy. After the IRE1 signaling pathway is activated, JNK phosphorylates the anti-apoptotic protein Bcl-2, which subsequently interacts with Beclin 1 under physiological conditions. This phosphorylation leads to the disruption of the Bcl-2/Beclin1 complex and allows Beclin-1 to trigger autophagy [50]. To investigate if the IRE1-JNK-Beclin 1 pathway was involved in the relationship between the ER stress response and autophagy, we examined the activities of p-JNK, p-Bcl-2, Beclin 1, and LC3-II after siRNAs transfection to ST cells to knock down endogenous IRE1. The knockdown of IRE1 decreased SADS-CoV N protein level and accumulation of LC3-II, accompanied by decreased expression of p-JNK and p-Bcl-2 in the SADS-CoV infected ST cells (Fig 10A). Class III PI3K, also named Vps34, plays an important role in autophagy induction by interacting with Beclin 1 [51]. Given the importance of the Vps34/Beclin 1 complex in autophagosome formation, we further investigated the role of Vps34/Beclin 1 complex in PLP2-TM induced autophagy. The results showed that PLP2-TM overexpression did not significantly affect the levels of Vps34 and Beclin 1 in Vero E6 (Fig 10B and 10D) and ST cells (Fig 10C and 10E). The effect of HA-PLP2-TM on the phosphorylation of Bcl-2 was examined owing to a crucial role of Bcl-2 phosphorylation in the interaction with Beclin 1 [52]. HA-PLP2-TM treatment dose independently up-regulated the phosphorylation of Bcl-2 and LC3-II proteins, but the expression level of Bcl-2 was not significantly changed in Vero E6 (Fig 10B and 10D) and ST (Fig 10C and 10E) cells. Furthermore, constructs lacking the 451–490 aa of PLP2-TM failed to up-regulate the phosphorylation of Bcl-2 and LC3-II proteins in Vero E6 (Fig 10F) and ST (Fig 10G) cells. Bcl-2 has been reported to function as a negative regulator of Vps34/Beclin 1 complex-dependent autophagy through binding with Beclin 1 [51]. We therefore investigated the effect of HA-PLP2-TM on the interaction of Beclin 1 and Bcl-2. Immunoprecipitation assays showed that HA-PLP2-TM overexpression significantly attenuated the interaction of Beclin 1 and Bcl-2 (Fig 10H). Thus, these data implied that SADS-CoV or PLP2-TM protein promoted ER stress-induced autophagy through the IRE1-JNK-Beclin 1 signaling pathway.

### Regulation of autophagy does not affect cell viability

In this study, we examined the influence of autophagy during SADS-CoV infection by pharmacological or siRNA alterations of autophagy. We found that the cell viability was not significantly changed by CCK-8 assay, which provided the basis for our further explore aiming to the relationship among autophagy and viruses (S2 and S3 Figs).

## Discussion

A growing number of evidences indicate autophagy can be affected by a variety of viruses [53–56]. SADS-CoV is a newly discovered coronavirus that can cause severe and acute diarrhea and rapid weight loss in piglets younger than 6-day-old [57]. However, there has been no study showing the interplay between cellular autophagy and SADS-CoV replication. In this study, we focused on revealing the interplay between SADS-CoV and host cells, in particular, the effects of cellular autophagy on the replication of SADS-CoV, and the underlying mechanisms.

SADS-CoV infection significantly increased the amount of single- or double-membrane vesicles (DMVs) in the perinuclear region of cultured cells (Fig 1A), which is consistent with that in several other coronaviruses, such as MHV, SARS-CoV-1, IBV, and Middle East respiratory syndrome coronavirus. This phenomenon is a hallmark of coronavirus replication and may potentially provide a platform for viral RNA synthesis [8,58–62]. During coronavirus infection, the formation of DMVs in the infected cells is usually associated with the autophagy pathway.

Furthermore, the conversion of LC3 was used to identify the formation of autophagic vesicles that is related to cellular autophagy. Accumulation of the exogenous protein GFP-LC3, as visualized by punctate fluorescence staining, represents a step in the cellular autophagy process. Subsequently, confocal immunofluorescence microscopy analysis showed that GFP-LC3 displayed many positive puncta in the cytoplasm of SADS-CoV-infected cells and that viral N protein was predominantly expressed in the cytoplasm (Fig 1C). Notably, LC3-II level did not increase in ST cells inoculated with UV-inactivated SADS-CoV, indicating that active replication of SADS-CoV was required for the induction of cellular autophagy (Fig 1D).

Recent studies have demonstrated that replication mechanisms may vary among different members of the Coronaviridae family [63]. The effect of autophagy on the replication of Coronaviruses is also different in various cells. For example, TGEV infection can induce mitochondrial autophagy (mitophagy) in IPEC cells mediated by DJ-1 (a multifunctional redox-sensitive protein), which enhances viral replication by reducing apoptosis [64]. Doxycycline (DOX) induces mitochondrial autophagy and promotes TGEV replication in porcine intestinal epithelial (IPEC-J2) cells [65]. Autophagy is also induced by TGEV infection in PK-15 and ST cells, and negatively regulates viral replication [41]. PEDV infects IPEC-J2 and Vero E6 cells. A study has shown that PEDV-induced autophagy enhances viral replication through PI3K/AKT/mTOR signaling pathway in IPEC cells [66]. On the contrary, rapamycin increases autophagy flux in IPEC-J2 cells, inhibits PEDV infection, and reduces PEDV-induced cell death [67]. Another study shows that autophagy benefits PEDV replication through autophagy regulators and RNA interference [68]. IBV can induce autophagy but does not require autophagy for replication [69]. The specific effect of autophagy induction on MHV infection is controversial. One study reported that MHV could utilize autophagy process to form DMVs for enhancing viral replication [59]. However, another study showed that autophagy was not important for MHV replication [70]. These observations suggest that replication mechanisms may differ among viruses of the same family and even for the same species. These differences may be related to differences of cell-type specificity or infection mechanisms. To understand the role of cellular autophagy in SADS-CoV replication, we examined if activation or inhibition of autophagy could affect SADS-CoV replication. First, the results indicated that regulation of autophagy with the pharmacological compounds, rapamycin or 3-methyladenine, increased or inhibited viral N protein expression and also enhanced or reduced viral yields (Fig 2). In addition, due to the important roles of ATG5 and LC3 played in inducing autophagosome formation [71], we further clarified the mechanisms if interruption of autophagy through silencing the ATG5 and LC3 proteins could affect viral production. The results demonstrated that knockdown of ATG5 or LC3 proteins significantly inhibited the expression of viral proteins as well as viral production (Fig 3). This result is similar to that in another coronavirus MHV. MHV can induce cellular autophagy, and ATG5 is required for maintaining viral replication at a normal level. Lipidation and membrane association of LC3 are dependent on ATG5, and these events are crucial for the formation of autophagic vesicles [59]. Taken together, these findings demonstrated that autophagy induction might benefit SADS-CoV replication.

During autophagy process, autophagosomes fuse with lysosomes, leading to the degradation of autophagic cargoes and cell substrates by lysosomal hydrolases [72]. The term “autophagic flux” is used to denote the dynamic process of autophagosome synthesis, the delivery of autophagic cargoes to the lysosome, and the degradation of autophagic cargoes inside of the lysosome. This tends to be a more reliable indicator of autophagic activity than measurements of autophagosome number. Interestingly, the autophagic response to different viruses is not always the same. The autophagic flux induced by some viruses is complete. For example, TGEV infection has been reported to enhance autophagic flux and induce complete autophagy [73]. However, some viruses were believed to inhibit the maturation of autophagosomes in virus infected cells. This was true for SARS-CoV-2, Coxsackievirus A16, hepatitis B virus and PRRSV, all of which can disrupt autophagosome trafficking to lysosomes, ultimately resulting in the accumulation of autophagosomes [74–77]. However, the exact details of how coronaviruses in general, and SADS-CoV in particular, intertwined with autophagy are still need to be further elucidated. In terms of lysosome-dependent signaling, the reduction of p62 protein expression and the transport of LC3 during virus infection are actually considered as the ultimate outcomes that mark complete autophagy [78]. In the present study, it was demonstrated that SADS-CoV infection induced autophagosomal maturation. Immunoblotting results showed that SADS-CoV infection decreased the expression of p62 protein (Fig 4A and 4B), indicating that SADS-CoV infection enhanced the autophagic flux in target cells. Moreover, by using a GFP-RFP-LC3 tandem construct to morphologically trace autophagic flux, the maturation of autophagosomes into autolysomes was assessed. The results indicated that the accumulation of autophagosomes may be due to *de novo* formation, but not the inhibition of their maturation (Fig 4C). These findings demonstrated that complete autophagic flux was triggered upon SADS-CoV infection. Additionally, lysosomal hydrolysis activity was inhibited by CQ, significantly reducing N protein expression and viral titers. The fusion of autophagosomes with lysosomes was inhibited by BAF A1, which reduced viral yields (Fig 5). Viral DMVs, resembling autophagosomes, may fuse with late endosomes or lysosomes, suggesting that vesicle acidification may play a role in viral replication or maturation of virions. For some coronaviruses, vesicle acidification is important for release of viral genome into the cytoplasm of the host cells during viral entry [79]. This finding was a direct indicator showing a complete autophagic process was induced by SADS-CoV infection.

An increasing number of studies have reported that viruses can induce autophagy by activating diverse pathways. For instance, autophagy can be induced in host cells through the triggering of EIF2AK2/PKR (eukaryotic translation initiation factor 2α kinase 2) expression by herpes simplex virus type 1 [80], the activation of the MAPK1/ERK2 (mitogen-activated protein kinase 1) by Epstein-Barr virus [81], the modulation of mTOR (mechanistic target of rapamycin) by IBDV [37], and the induction of ER stress by hepatitis C virus [82] and IBV [83]. The ER serves as an important sensor of cellular stress. Coronavirus replication is structurally [84] and functionally [61] associated with the ER, and has been shown to induce ER stress and activate all three branches of the UPR [29,85,86]. Therefore, it was hypothesized that SADS-CoV infection might induce autophagy through ER stress-associated UPR signaling pathway. In this study, it was found that SADS-CoV infection up-regulated GRP78 and activated all three UPR pathways following SADS-CoV infection *in vitro* (Fig 6A–6C). The activation of multiple UPR signaling pathways by a viral infection has been reported in other coronaviruses, such as SARS-CoV-1, MHV, IBV, and HCoV-OC43 [87–90]. Importantly, significant autophagy and ER stress in SADS-CoV-infected ileum tissue *in vivo* were observed (Fig 6E and 6F), indicating that autophagy and ER stress was involved in the pathogenesis of SADS-CoV *in vivo*. Moreover, using RNAi and inducer or inhibitor of ER stress, we demonstrated that SADS-CoV induced autophagy was dependent on ER stress.

Viruses differ in their ability to activate the three arms of the UPR transmembrane sensors: PERK, IRE1, and ATF6. Some viruses activate all sensors, while some viruses activate only one or two during infection [91]. In this study, all three UPR branches were activated both *in vitro* and *in vivo* following SADS-CoV infection. However, each pathway of the three UPR branches has a different role in viral replication. The ATF6 pathway does not modulate SADS-CoV replication, based on the results of siRNA interference and overexpression assays (Fig 7E–7H). The PERK-EIF2S1 branch is strongly associated with viral replication and pathogenesis [92,93]. Here, we demonstrated that the SADS-CoV-induced PERK-EIF2S1 pathway activation primarily accounted for the increased EIF2S1 phosphorylation and negatively regulated SADS-CoV replication. Generally, global translation inhibition induced by phosphorylated EIF2S1 represents a vital host cellular antiviral response, which is detrimental to viral replication. Similar to this finding, the replication of TGEV and PEDV is also suppressed by PERK-EIF2S1 signaling [94,95]. In contrast, for IBV and MHV, replication was not markedly affected by activated PERK-EIF2S1 [89,96]. Thus, PERK-EIF2S1 signaling has diverse roles in different coronavirus infections, and the detailed mechanism of inhibited SADS-CoV replication needs to be further clarified. The specific roles of the three UPR branches in autophagy and viral replication are not the same, although SADS-CoV infection induced all three UPR signaling branches. In this study, it was found that among the three ER stress sensors, only IRE1, but not ATF6 or PERK-EIF2S1, was required for autophagy induction during SADS-CoV infection (Fig 7). These results are in line with previous studies demonstrating an intriguing link between the UPR and autophagic vacuole formation [46,97–99]. This also demonstrated that the IRE1 signaling pathway was required for the activation of autophagy under the UPR. PERK-EIF2S1 and ATF6 pathways are not needed for the activation of autophagy [46]. The data in the present study also suggested that the IRE1 pathway regulated autophagic events independently, thereby playing a crucial role in supporting SADS-CoV replication. This is consistent with the previous findings [29], showing that *alphacoronavirus* TGEV replication was suppressed following the knockdown of IRE1. In addition, all three UPR pathways were activated by TGEV infection *in vitro* and *in vivo*.

In recent years, several proteins of coronaviruses were shown to induce the formation of double-membrane structures, such as the nsps 2, 3, 4, and 6 [100]. In this study, we presented evidence that the expression of PLP2-TM alone was capable of activating complete autophagy and ER stress (Figs 8 and 9), assigning a novel function to this multifunctional viral protein that has been known to act as viral protease, deubiquitinating enzyme, and IFN antagonist [101]. ER stress signaling is a cellular adaptive mechanism that occurs in response to the disruption of ER homeostasis. GRP78 is a well characterized ER chaperone and also a master modulator of UPR. GRP78 can interact with unfolded proteins through its C-terminal substrate-binding domain, which is intensively regulated by a conformational change that depends on ATP occupation of its NBD [102]. Our results showed that the membrane-anchored coronavirus papain-like protease PLP2 domin (and its homologues) can interact with GRP78, and the PLP2-TM^F451-L490^ domain of SADS-CoV served for the binding with SBD of GRP78 (Fig 9). ER stress triggers autophagy through PERK/EIF2S1, IRE1/JNK, or potentially cleavage of EIF2S1 by caspase [103]. The IRE1/JNK pathway participates in the release of Beclin 1, which in turn triggers the formation of autophagosomes and the conversion of LC3-II, thus promoting autophagy by alleviating Beclin 1 inhibition through downregulation of Bcl-2 [104]. In this study, a western blotting analysis revealed that the expressions of p-JNK and p-Bcl-2 reduced upon knockdown of IRE1 in SADS-CoV infected Vero E6 and ST cells (Fig 10A). Further, we found that PLP2-TM expression led to the phosphorylation of Bcl-2 and the conversion of LC3-II (Fig 10B–10E). The phosphorylation of Bcl-2 and the conversion of LC3- II were interrupted if the 451–490 aa of PLP2-TM were deleted (Fig 10F–10G). Beclin 1 contains a Bcl-2-binding domain, and Bcl-2 has been reported to negatively regulate autophagosome formation through binding to Beclin 1 [51]. We therefore investigated the effect of PLP2-TM on the interaction between Bcl-2 and Beclin 1. PLP2-TM overexpression indeed inhibited the association of Beclin 1 with Bcl-2 (Fig 10H). However, our data showed that SADS-CoV and PLP2-TM did not appear to significantly affect the level of Beclin 1 expression. In agreement with previous results, Sir et al. [105] have reported that neither HBx nor HBV has a significant effect on Beclin-1 induction.

**Fig 11:**
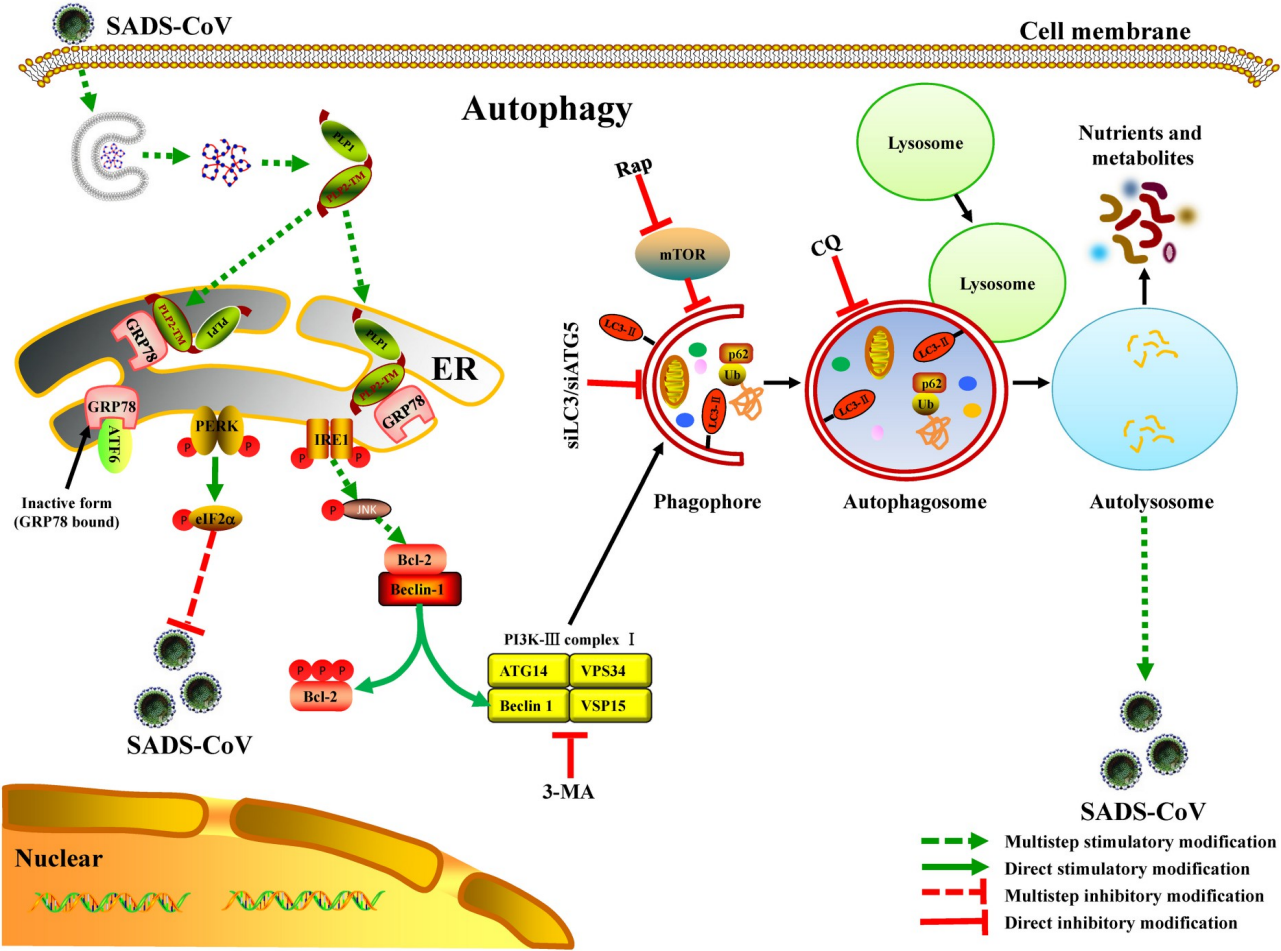
Graphical illustration of the IRE1-JNK-Beclin 1 signaling pathway involved in SADS-CoV-induced autophagy via PLP2-TM and GRP78 interaction.

In this study, we reported that the IRE1-JNK-Beclin 1 pathway regulated autophagy during SADS-CoV infection. We showed that SADS-CoV-induced autophagy facilitated viral replication. Moreover, we provided the first evidence that the papain-like protease alone, through their transmembrane anchors, activated complete autophagy by interacting with GRP78 (Fig 11). A precise understanding of the molecular mechanisms involved in the interplay between SADS-CoV and autophagy is still incomplete. Therefore, further work for revealing the immunological function of autophagy, and its role in the immune escape of SADS-CoV, is of importance. This information may provide benefits for controlling viral infection and useful insights for the development of novel antiviral strategies against SADS-CoV infection.

## Materials and methods

### Ethics statement

The animal experiments were approved by Harbin Veterinary Research Institute. The Animal Ethics Committee approval number was Heilongjiang-AUP-20190801.

### Cells and viruses

Vero E6, ST, HEK 293T (American Type Culture Collection, ATCC), and IPI-2I (Our Lab) cells cultured in Dulbecco’s Modified Eagle’s Medium (DMEM; Life Technologies; 11995) supplemented with 100 mg/mL streptomycin, 100 units/mL penicillin, and 10% fetal bovine serum (Gibco; Life Technologies; 10099–141) at 37°C in a 5% CO_2_ environment. SADS-CoV was isolated from clinical samples with diarrheal symptoms from pig herds in Guangdong Province of China, and kept in our laboratory. Specimens from piglets with signs of severe diarrhea and a high mortality were collected from February to August, 2017 on four SADS-CoV positive farms.

### UV-inactivated SADS-CoV

To create replication-deficient virus, the supernatant containing SADS-CoV was ultraviolet (UV)-irradiated on ice at 100 μJ/cm^2^ for 60 min using a CL-1000 crosslinker. Vero E6 cells were incubated with the UV-inactivated SADS-CoV supernatant for 1 h in an incubator. Then, the supernatant was replaced with DMEM containing 1 μg/mL trypsin and maintained for 48 h. The inactivation efficiency of cells that were infected with UV-inactivated SADS-CoV was measured using an immunofluorescence assay with a specific anti-viral protein N antibody.

### Recombinant plasmids and reagents

A series of vectors were constructed to satisfy the experimental needs, and all plasmids were verified by sequencing. To obtain high expression of PLP2-TM homologues of coronaviruses in eukaryotic cells, the codon usage of PLP2-TM homologues (1,588–2,120aa of pp1a of SADS-CoV, GenBank accession no. MG557844.1; 1563-2408aa of pp1a of SARS-CoV-2, GenBank accession no. QVM14311.1; 1,239–1,908aa of pp1a of IBV, GenBank accession no. NP_040829.1; 702-1657aa of pp1a of PDCoV, GenBank accession no. QUM93286.1) and nsp4 gene of SADS-CoV were optimized on the basis of the human codon usage frequency. The target genes were cloned into pCAGGS-HA with in-frame fusion with N-terminal HA tag. The cDNAs for swine ATF6 (GenBank accession no. XM_021089510) and GRP78 (GenBank accession no. NM_005347) protein were cloned into the pCMV-Myc and pCMV-Flag vectors to generate the recombinant plasmids pMyc-ATF6 and pFlag-GRP78, respectively. The plasmids pGFP-LC3, pGFP-RFP-LC3 [73], and pHA-EIF2S1 [29] were described previously. The anti-SADS-CoV N monoclonal antibody derived from mouse was kept in our laboratory. The antibodies against LC3 (ab51520), ATG5 (ab108327), p62 (ab56416), GRP78 (ab21685), p-IEF2S1 (Ser 51) (ab32157), p-IRE1 (Ser 724) (ab48187), ATF6 (ab122897), p-JNK (ab124956), p-Bcl-2 (ab218123), Vps34 (ab124905), Beclin 1 (ab210498), total EIF2S1 (ab5369), total IRE1 (ab37073), total Bcl-2 (ab32124), total JNK (ab179461), and HA (ab9110) were purchased from Abcam (Cambridge, MA). The antibodies against Flag (F1804), GAPDH (G9545), Myc (M4439), and mouse lgG (whole molecule)-tetramethyl rhodamine isothiocyanate (TRITC) antibody produced in goat (T5393) were purchased from Sigma-Aldrich. Rapamycin (R0395), 3-methyladenine (M9281), chloroquine diphosphate salt (C6628), 4-PBA (P21005), DTT (D9779), salubrinal (324897), and 4μ8C (412512) were purchased from Sigma-Aldrich (St. Louis, MO, USA).

### Transmission electron microscopy (TEM)

The treated cells were washed three times with phosphate-buffered saline (PBS) and then fixed with 2.5% glutaraldehyde in 0.1 M phosphate buffer (pH = 7.4) overnight, and followed by TEM observation [106]. Autophagosomes were defined as double or single membrane vesicles of 0.3–2.0 μm in diameter.

### Cell culture, virus infection, and treatments with chemicals

Vero E6 and ST cells were infected with SADS-CoV (MOI = 0.1) or mock infected with PBS. After 1 h of incubation at 37°C, unbound viruses were removed from cells by three washes with PBS, and the cells were cultured in DMEM supplemented with 1 μg/mL trypsin at 37°C for different time courses.

In accordance with the requirements of different experiments, the Vero E6 and ST cells were pretreated with different concentrations of chemicals or the same volume of DMSO, and followed by inoculation with SADS-CoV (MOI = 0.1). After incubation for 1 h, the supernatants were removed and replaced with cell culture media containing different doses of the chemicals. The cells were then cultured at 37°C for the indicated time points until the necessary samples were harvested for further experiments.

### Confocal fluorescence microscopy

Vero E6 cells were seeded on microscope slide coverslips, which were set in 35-mm diameter dishes, and grown to ~80% confluence. Then, the cells were transfected with the indicated plasmids according to the manufacturer’s instructions. The cells were infected with SADS-CoV or treated with drugs after transfecting with the indicated plasmids for 24 h. The cells were fixed with precooled 4% paraformaldehyde (Sigma-Aldrich; 16005) in PBS for 30 min and permeabilized using 0.1% Triton X-100 (Sigma-Aldrich; T8787) and in 2% BSA (Beyotime; ST023) in PBS for 1 h. Then, the cells were incubated with different primary antibodies for 6–8 h at 37°C. After three washes with PBS-Tween-20 (PBST containing 0.05% Tween-20 (Sigma-Aldrich; P1379), the cells were incubated with appropriate secondary antibodies (Alexa 488, 594 or 647-conjugated) for an additional 1 h. Nuclear DNA was stained with 4′,6-diamidino-2-phenylindole (DAPI) for 15 min and then washed with PBS three times (5 min per time). Finally, the cells were directly observed under a LSM880-ZEISS confocal laser scanning microscope equipped with Fast Airyscan (Zeiss).

### Live cell imaging

Vero E6 cells were cultured to ~70% confluence in 30-mm culture dishes with 20-mm coverslip inserts, and were transfected with GFP-RFP-LC3 by using Lipofectamine 3000 (Invitrogen, L3000015). At 24 h post-transfection, cells were mock-infected or infected with SADS-CoV at an MOI of 0.1. After 1 h of absorption, cells were observed by long-term live cell imaging with the RFP and GFP channels to produce time-lapse movies. By using this confocal imaging, three-dimensional data could be captured by collecting two-dimensional images in the XY plane at a range of Z-plane settings. Due to low levels of light, the three-dimensional data were captured in real time without damaging the cells, ensuring autophagic processes were recorded if occurred. After capturing images, the Volocity analysis tool (Ultra View Vox, PerkinElmer) was employed to extract data and quantify cellular processes. Images and data were then transferred to a video program for display.

### RNA interference

The siRNAs against ATG5, LC3, GRP78, ATF6, IRE1, and control scrambled siRNA were designed by the RiboBio (Guangdong, China), and their sequences were listed in Table 1. Cells were seeded in a 6-well plate and grown to 40~50% confluence. The siATG5, siLC3, siGRP78, siEIF2S1, siATF6, sire1, and control scrambled siRNA were transfected into cells using Lipofectamine RNAiMAX transfection reagent (Invitrogen, 13778150) according to the manufacturer’s instructions. At 48 h post-transfection, cells were infected with SADS-CoV and harvested at the indicated time points for protein and virus titration analyses.

**Table 1:** The siRNA target sequences for ATG5, LC3, GRP78, ATF6 and IRE1 protein expression in ST cells.

Target	siRNA sequence	Targeted sequences
ATG5	1st	GCTCTTCCTTGGAACATCA
	2nd	CCATCAATCGGAAACTCAT
LC3	1st	ATTCCTGTACATGGTCTAT
	2nd	GATTCCTGTACATGGTCTA
GRP78	1st	GGAGCGCATTGATACTAGA
	2nd	GAGGTAAACTTTCCTCTGA
ATF6	1st	CAGACAGTACCAACGCTTA
	2nd	CAGTCTCGCAAGAAGAAGA
IRE1	1st	CCATCATCCTGAGCACCTT
	2nd	CCAGAAGGAACTAGAGAAATT

### SDS-PAGE and western blotting

Whole cell lysates were prepared at different time points after infection and transfection using RIPA lysis buffer (Sigma-Aldrich, R0278) containing 1 mM phenylmethanesulfonyl fluoride (PMSF; Beyotime, ST506-2) in accordance with the manufacturer’s instructions. The total protein concentration was measured using a bicinchoninic acid protein assay kit (Thermo Fisher Scientific, 23225). Equal amounts of total proteins were analyzed with SDS-PAGE and then transferred onto nitrocellulose membranes (Pall, 66485). After blocking with 5% skim dry milk for 1 h at 37°C, the membranes were incubated with different primary antibodies for 6–8 h at 4°C, and followed by incubation with IRDye 800CW goat anti-mouse lgG (H+ L) (1:10,000) (926–32210; LiCor BioSciences) and IRDye 680RD goat anti-rabbit lgG (H+ L) (1:10,000) (926–68071; LiCor BioSciences) at 37°C for 1 h, and thereafter the blots were visualized using an Odyssey infrared imaging system (LiCor BioSciences). Quantification of band intensities by densitometry was carried out using the Image J software.

### Virus titration

Vero E6 and ST cells grown in 6-well culture plates were treated with Rap, 3MA, CQ, ISRIB, 4μ8C, salubrinal, or DMSO, and then infected with SADS-CoV at an MOI of 0.1 for the indicated time courses, or transfected with siATG5, siLC3, siGRP78, siEIF2S1, siATF6, or siIRE1 for 48 h, and then infected with SADS-CoV at an MOI of 0.1. At 36 hpi, the culture supernatants were collected. To assess the viral titers, the Vero E6 cells were seeded into 96-well plates, and the medium was removed after the cells were incubated with viruses for 1 h in an incubator. Then, the viral inoculum was removed and the infected cells were washed three times with PBS (pH = 7.4) and refed with DMEM containing 1 μg/mL trypsin. Viral titers were calculated as 50% tissue culture infectious doses (TCID_50_) according to the Reed-Muench method [107].

### Experimental infection of piglets and immunohistochemistry (IHC)

Twelve 3-day-old specific pathogen-free (SPF) piglets were randomly divided into two groups. The SPF piglets in group 1 were orally inoculated with SADS-CoV of 5 × 10^4^ TCID_50_. SPF piglets in group 2 were inoculated with DMEM, serving as uninfected controls. After inoculation, the piglets were observed and recorded three times daily for clinical symptoms of vomiting, diarrhea, lethargy, and overall body condition. All of the piglets were euthanized at the end of this study, which was terminated at 36 hpi. The small intestine samples from the piglets were collected for IHC analyses. Representative sections of the ileal tissues were fixed with 4% paraformaldehyde and stored in 70% ethanol at 4°C. The IHC assay was performed based on previous description [29]. Slides were incubated with mAb 3E9 (1:50) at 4°C overnight and subsequently hybridized with HRP-labelled goat anti-mouse lgG (Sigma-Aldrich, AP308P) for 1 h. Immunocomplexes were detected using the 3,3’-diaminobenzidine liquid substrate system.

### Immunoprecipitation

Immunoprecipitation was performed as described previously [108]. Briefly, cells were lysed in IP lysis buffer (87788, Thermo) containing 1 mM PMSF and 1 mg/mL protease inhibitor cocktail (04693132001; Roche) at 4°C for 30 min. After being precleared with Protein A/G agarose beads for 2 h at 4°C, lysates were incubated with the indicated primary antibody at 4°C overnight, and followed by incubation with Protein A/G agarose beads for 2 h. The beads were washed five times with lysis buffer. Immunoprecipitated proteins were separated on SDS-PAGE and transferred to NC membranes for western blot analysis.

### Cell viability assay

The cell viability assay was performed using the cell counting kit-8 (CCK-8) (CK04; Dojindo) according to the manufacture’s instructions.

### Statistical analysis

All results shown in the figures were presented, where appropriate, as means and standard deviations (SD) from the results of three independent experiments were analyzed with GraphPad Prism (GraphPad Software, Inc.). Differences were considered significant if the *P* value < 0.05.

## Supporting information

S1 Movie Autolysosome formation in SADS-CoV-infection cells. Vero E6 cells are transfected with plasmid expressing GFP-RFP-LC3 for 12 h, and the cells are infected with SADS-CoV at an MOI of 1. After absorption for 1 h, cells are observed over time by live cell imaging with RFP and GFP channels to produce time-lapse movies. The present data are derived from one of three independent experiments.
(AVI)

S2 Movie Autolysosome formation in mock-infection cells. Vero E6 cells are transfected with plasmid expressing GFP-RFP-LC3 for 12 h, and the cells are mock infected with basic DMEM. After absorption for 1 h, cells are observed over time by live cell imaging with RFP and GFP channels to produce time-lapse movies. The present data are derived from one of three independent experiments.
(AVI)

S1 Fig The membrane-associated papain-like protease PLP2 of SADS-CoV, but not nsp4 acts as an autophagy-inducing protein. Vero E6, HEK293T, and ST cells are transfected with plasmids expressing HA-nsp4, HA-PLP2-TM, or empty vector for 36 h. The cells treated with 250 nM rapamycin serve as a positive control for the induction of autophagy. The expression levels for each protein are analyzed by western blotting analysis with the indicated antibodies (Right panel).
(TIF)

S2 Fig Cell viability is detected with a CCK-8 assay after treatments with Rap, 3MA, CQ, BAF A1, Salubrinal, and 4μ8c. Cell viability is detected by CCK-8 assay after treatments with Rap (500 nM), 3MA (2 mM), CQ (20 mM), BAF A1 (20 nM), Salubrinal, and 4μ8C (100 μM) in Vero E6 and ST cells for 48 h. Light absorption at 450 nm is recorded and expressed as a percentage of relative cell viability, and the values are represented as the mean ± SD (n = 3). Significant differences are assessed by one-way ANOVA. “ns” means no significant difference compared to control, *P* > 0.05.
(TIF)

S3 Fig siRNA treatment does not affect cell viability. Cell viability is detected by CCK-8 assay after transfection with siRNAs in ST cells for 48 h. Light absorption at 450 nm is recorded and expressed as a percentage of relative cell viability, and the values are represented as the mean ± SD (n = 3). Significant differences are assessed by one-way ANOVA. “ns” means no significant difference compared to control, *P* > 0.05.
(TIF)
