# Supplementary figures and images for: Autophagy is induced by swine acute diarrhea syndrome coronavirus through the cellular IRE1-JNK-Beclin 1 signaling pathway after an interaction of viral membrane-associated papain-like protease and GRP78
Source: PLoS Pathog. 2023 Mar 8;19(3):e1011201. doi: 10.1371/journal.ppat.1011201 (PMC9994726; doi:10.1371/journal.ppat.1011201)

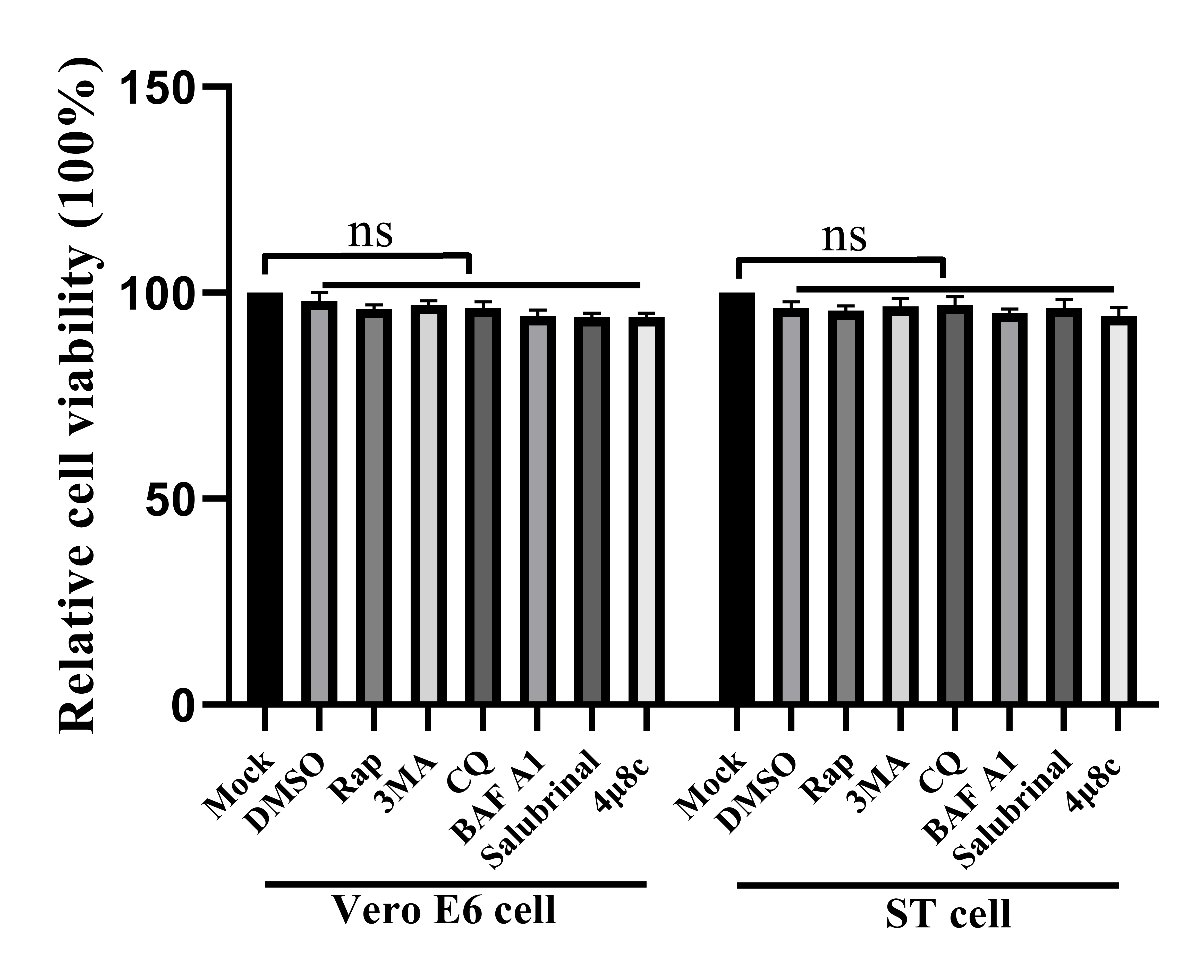

Supplement: S2 Fig — Cell viability is detected by CCK-8 assay after treatments with Rap (500 nM), 3MA (2 mM), CQ (20 mM), BAF A1 (20 nM), Salubrinal, and 4μ8C (100 μM) in Vero E6 and ST cells for 48 h. Light absorption at 450 nm is recorded and expressed as a percentage of relative cell viability, and the values are represented as the mean ± SD (n = 3). Significant differences are assessed by one-way ANOVA. “ns” means no significant difference compared to control, P > 0.05. (TIF) [file ppat.1011201.s004.tif]

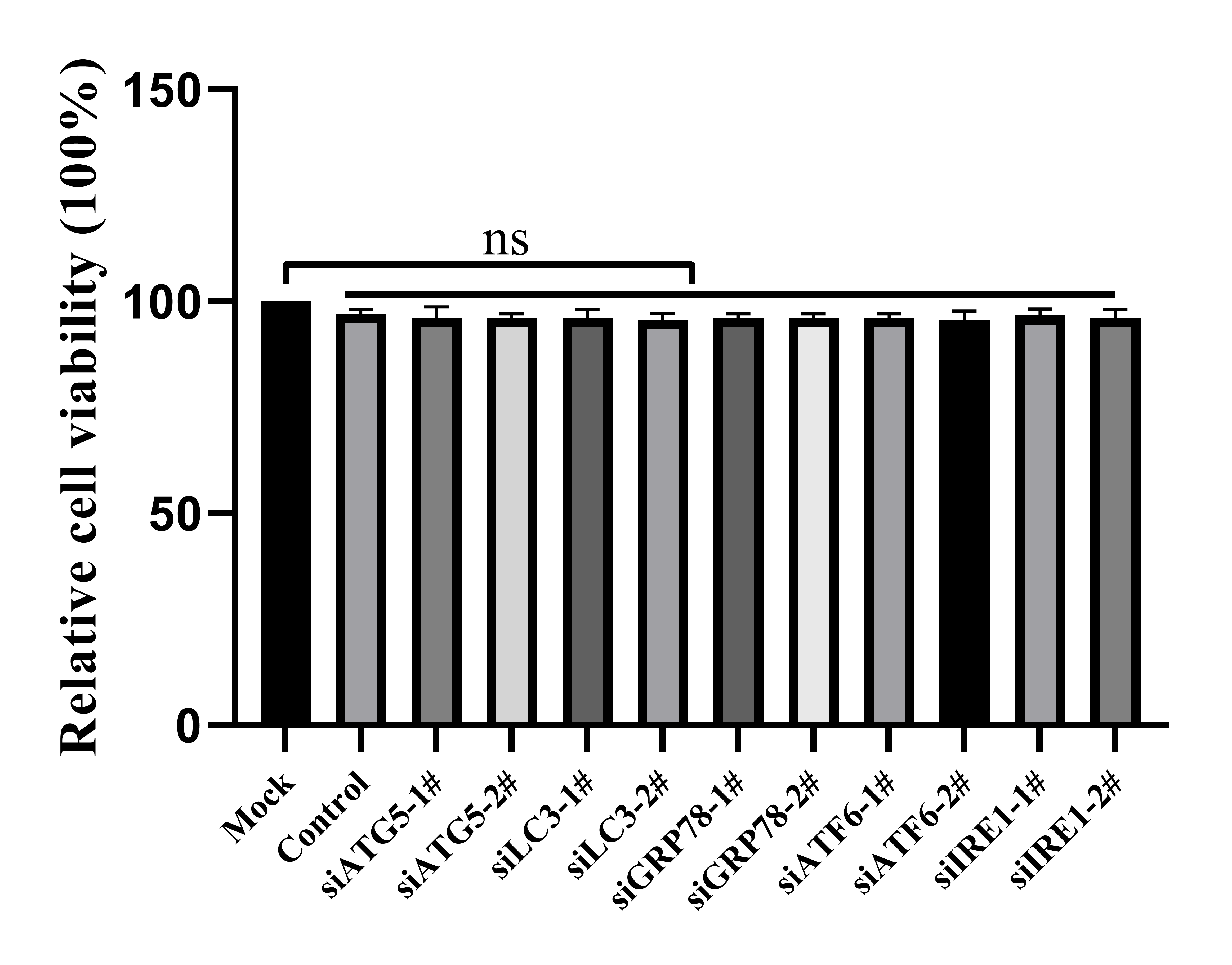

Supplement: S3 Fig — Cell viability is detected by CCK-8 assay after transfection with siRNAs in ST cells for 48 h. Light absorption at 450 nm is recorded and expressed as a percentage of relative cell viability, and the values are represented as the mean ± SD (n = 3). Significant differences are assessed by one-way ANOVA. “ns” means no significant difference compared to control, P > 0.05. (TIF) [file ppat.1011201.s005.tif]
